# Motility and Chemotaxis Mediate the Preferential Colonization of Gastric Injury Sites by *Helicobacter pylori*

**DOI:** 10.1371/journal.ppat.1004275

**Published:** 2014-07-17

**Authors:** Eitaro Aihara, Chet Closson, Andrea L. Matthis, Michael A. Schumacher, Amy C. Engevik, Yana Zavros, Karen M. Ottemann, Marshall H. Montrose

**Affiliations:** 1 Department of Molecular and Cellular Physiology, University of Cincinnati, Cincinnati, Ohio, United States of America; 2 Department of Microbiology and Environmental Toxicology, University of California at Santa Cruz, Santa Cruz, California, United States of America; University of Illinois, United States of America

## Abstract

*Helicobacter pylori* (*H. pylori*) is a pathogen contributing to peptic inflammation, ulceration, and cancer. A crucial step in the pathogenic sequence is when the bacterium first interacts with gastric tissue, an event that is poorly understood *in vivo*. We have shown that the luminal space adjacent to gastric epithelial damage is a microenvironment, and we hypothesized that this microenvironment might enhance *H. pylori* colonization. Inoculation with 10^6^ *H. pylori* (wild-type Sydney Strain 1, SS1) significantly delayed healing of acetic-acid induced ulcers at Day 1, 7 and 30 post-inoculation, and wild-type SS1 preferentially colonized the ulcerated area compared to uninjured gastric tissue in the same animal at all time points. Gastric resident *Lactobacillus* spp. did not preferentially colonize ulcerated tissue. To determine whether bacterial motility and chemotaxis are important to ulcer healing and colonization, we analyzed isogenic *H. pylori* mutants defective in motility (Δ*motB*) or chemotaxis (Δ*cheY*). Δ*motB* (10^6^) failed to colonize ulcerated or healthy stomach tissue. Δ*cheY* (10^6^) colonized both tissues, but without preferential colonization of ulcerated tissue. However, Δ*cheY* did modestly delay ulcer healing, suggesting that chemotaxis is not required for this process. We used two-photon microscopy to induce microscopic epithelial lesions *in vivo*, and evaluated accumulation of fluorescently labeled *H. pylori* at gastric damage sites in the time frame of minutes instead of days. By 5 min after inducing damage, *H. pylori* SS1 preferentially accumulated at the site of damage and inhibited gastric epithelial restitution. *H. pylori* Δ*cheY* modestly accumulated at the gastric surface and inhibited restitution, but did not preferentially accumulate at the injury site. *H. pylori* Δ*motB* neither accumulated at the surface nor inhibited restitution. We conclude that bacterial chemosensing and motility rapidly promote *H. pylori* colonization of injury sites, and thereby biases the injured tissue towards sustained gastric damage.

## Author Summary

*H. pylori* is a disease-causing bacterium that commonly infects the human stomach in both developed and underdeveloped countries. Infected individuals can develop digestive diseases, including stomach inflammation, peptic ulcer, and cancer. There has been only limited investigation into the events when *H. pylori* first interacts with stomach tissue. Using anesthetized mice in which we have induced microscopic damage to the stomach surface, we find that *H. pylori* is able to rapidly detect and navigate towards this damage site. Within minutes, bacterial accumulation slows repair of the damage. This is the earliest event of *H. pylori* pathogenesis that has been reported *in vivo*. We further define that this pathology is due to the bacterial accumulation at damage sites and that this also occurs in a model of larger stomach damage (ulceration). The broader implications of our work are that even sub-clinical insults to the stomach that occur in daily life (damage from grinding of food, ingestion of alcohol, taking an aspirin) can potentially attract *H. pylori* and not only slow repair of any existing damage, but maybe also provide an initiation site that can start the pathogenic sequence of stomach disease caused by *H. pylori*.

## Introduction

*H. pylori* infection promotes gastritis, gastric ulceration, and gastric cancer [1]. The mechanisms of early *H. pylori* interaction with gastric tissue have not been explored, but are essential for successful colonization and ultimately the disease consequences of *H. pylori* infection. *H. pylori* is believed to migrate through the gastric environment by chemotaxis, using its multiple flagella [2], [3]. Indeed, motility and chemotaxis have been shown to lower the dose of *H. pylori* needed to establish an infection [2], [3]. *In vitro*, *H. pylori* motility responds to various conditions, including CO_2_, urea/ammonium, arginine, bacterial energy status and low pH [4]–[9]. Additionally, *H. pylori* has been shown to use gradients that are sensitive to pH to localize close to the gastric epithelium *in vivo* [7]. We and others have shown that damage to the gastric epithelium (commonly produced by alcohol, salt intake, smoking, and non-steroidal anti-inflammatory drugs usage) can affect the luminal microenvironment adjacent to tissue [10]–[13]. We were thus curious whether *H. pylori* can sense and respond to these damage-induced microenvironments using chemotaxis, and whether these regions might serve as preferential colonization sites.

*H. pylori* uses largely canonical chemotaxis signal transduction to promote chemotaxis [4]. *H. pylori* senses its environment through four chemoreceptors TlpA, TlpB, TlpC, and TlpD that detect conditions including pH, autoinducer 2, CO_2_, arginine, urea/NH_4_ and molecules that lead to bacterial energy generation [4]–[6], [8], [9], [14], [15]. Ligand interactions with chemoreceptors control the direction of flagellar motor rotation *via* the Che family of proteins [4]. The most downstream member of the Che protein family, CheY, directly interacts with the flagellar motor to control flagellar rotational direction [4], [16], [17]. Generation of torque for flagellar rotation is regulated by members of the Mot family of proteins, MotA and MotB [4]. Isogenic mutant strains lacking the flagellar motor protein MotB (Δ*motB*) retain wild-type flagellar structure (Fla^+^), but the Mot^−^ flagella are non-functional [2]. *H. pylori* lacking the *motB* gene have no motility and a substantially reduced ability to colonize the stomach initially and to multiply to full bacterial loads [2], [4]. Mutants lacking CheY (Δ*cheY*) retain functional flagella (Mot^+^) but cannot change the direction of flagellar rotation, and thus are non-chemotactic (Che^−^) [3], [4], [18]. Δ*cheY* swim with fewer direction changes as compared to wild-type *H. pylori* [19], [20].

*In vivo*, chemotaxis promotes multiple aspects of *H. pylori* gastric colonization. Che^−^ *H. pylori* similarly show a reduced ability to colonize the stomach, particularly at early time points [3], [18], [21], [22]. In addition, they lose the ability to locate close to the epithelial surface and deep in the antral gastric glands [3], [21]. Thus, evidence supports that chemotaxis aids colonization, but whether it is directed toward sites of damage is not known.

In the present study, we test the role of *H. pylori* chemosensing and motility in response to damaged tissue *in vivo*, using acetic-acid ulcer induction as a long term injury/repair model, and then extend results to short term injury/repair using two-photon induced gastric epithelial damage.

## Results

We first confirmed the motility of wild-type *H. pylori* (Sydney Strain 1: SS1) used in this study by tracking individual fluorescently labeled *H. pylori* using a fast-scan confocal microscope (Movie S1). As shown in Figures S1 and S2, fluorescently labeled wild-type *H. pylori* (n = 6 experiments) had motility properties (swimming in straight or arced lines at mean velocity 25.5±0.7 µm/sec, number of stopping: 0.13±0.05 per sec, frequent changes in speed, slowed motility at pH 5 and pH 4) consistent with previous findings [19], [20], [23]. Results from fluorescently labeled *H. pylori* were indistinguishable from unlabeled *H. pylori* (velocity: 28.5±1.1 µm/sec, number of stopping: 0.15±0.06 per sec, n = 6).

### Ulcer repair is inhibited rapidly by *H. pylori*

**Figure 1:**
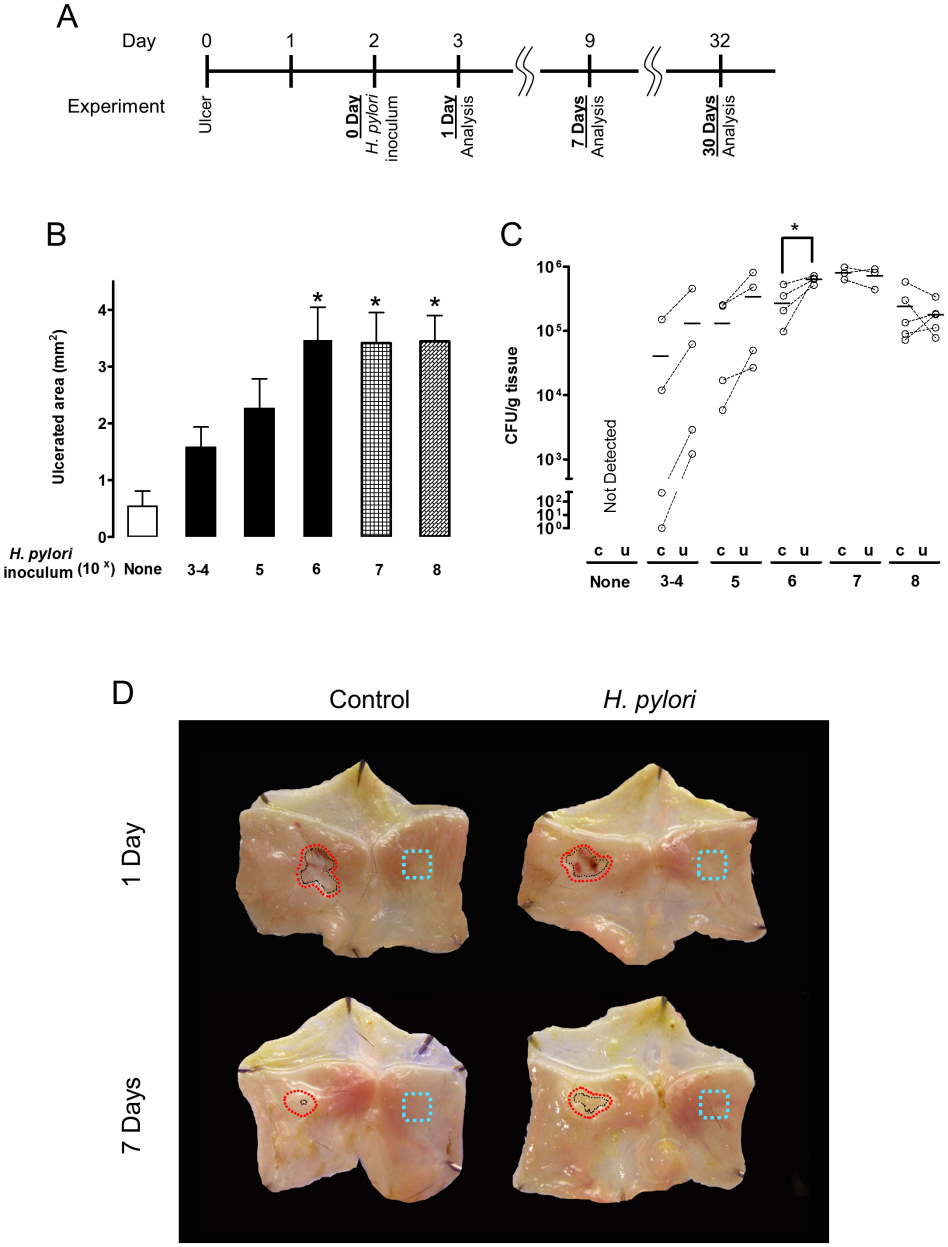
Effect of different amounts of *H. pylori* inoculation on gastric ulcer healing. (A) Schematic of experimental timeline, reconciling the potential confusion of counting days from ulceration *versus* days after *H. pylori* inoculation. A single gavage of 1×10^3^–10^8^ *H. pylori* was performed 2 days after ulcer induction. Gastric ulcer size (B) or *H. pylori* CFU (C) was measured 7 Days post-inoculum. Mean ± SEM (n = 4–5). *, p<0.05 vs. uninfected control (none *H. pylori*). (D) Gross morphology at experimental day 3 and 9 after ulceration (Day 1 or Day 7 after inoculation with 10^6^ *H. pylori*). Control tissue was uninfected. Black *dotted lines* indicate ulcer crater (macroscopic area lacking epithelium that is measured by digital caliper to evaluate ulcer size). Red *dotted lines* or blue *dotted lines* shows the location that collected for *H. pylori or* RT-PCR analysis as ulcerated area or intact area, respectively.

**Figure 2:**
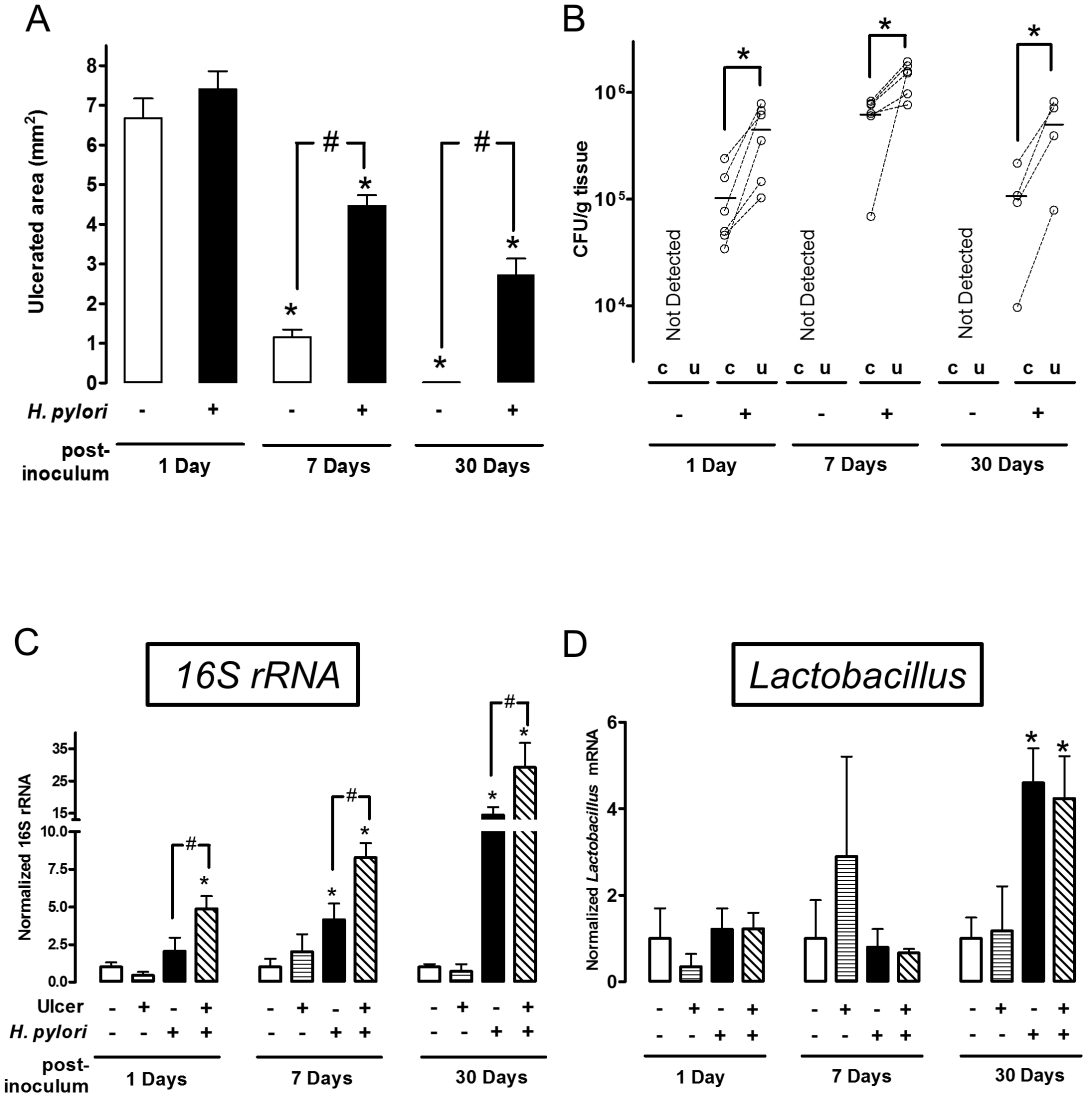
Time course of bacterial colonization and *H. pylori* effects on gastric ulcer healing. Gastric ulcer was induced by topical application of acetic acid to the exterior surface of the surgically exposed stomach. Where indicated (*H. pylori* +) a single gavage of 1×10^6^ *H. pylori* was performed 2 days after ulcer induction. Tissue was evaluated 1 (n = 6), 7 (n = 6) or 30 (n = 4) Days after *H. pylori* inoculation. (A) Gastric ulcer size was measured by calipers. Mean ± SEM. *, p<0.05 vs. Day 1; ^#^, p<0.05 vs. no *H. pylori* control group. (B) Harvested ulcerated (u) or non-ulcerated control (c) gastric tissue was collected as depicted in Figure 1D, homogenized and *H. pylori* cultured on Columbia blood agar plate to obtain CFU. Data are presented as CFU/g tissue, with lines connecting tissue from the same animal to indicate trends. Significant difference at *, p<0.05 vs. intact region. PCR Detection of 16S rRNA (C: total bacteria) or Lactobacillus (D), and data shown as fold change normalized to non-ulcerated control region of uninfected group (ulcer −, *H. pylori −*). mean ± SEM. *, p<0.05 vs. non-ulcerated control.

Acetic acid-induced ulcer models are well established and resemble human ulcers in terms of both pathological features and healing process [24]. Murine gastric ulcers were induced by serosal topical application of acetic acid to the exterior of the intact stomach corpus, then two days later, when ulcer size is maximal, wild-type SS1 *H. pylori* was gavaged into the ulcerated stomach. We evaluated both ulcer size and *H. pylori* colonization at days 3, 9 and 32 after ulcer induction (which are respectively, Day 1, 7, and 30 after inoculation; as shown in the Figure 1A diagram). Wild-type *H. pylori* delayed ulcer healing 7 Days after inoculation, in a dose dependent manner, as compared to almost complete ulcer healing in the absence of *H. pylori* over the same time interval (Figure 1B and 1D). Inoculation with ≥10^6^ *H. pylori* resulted in reproducible detection of colony forming units (CFU) from the small ulcerated area (Figure 1C) as well as significantly impaired ulcer healing, so was used as the routine inoculum. Looking at a more extensive time course, ulcer size diminished in the uninfected control group approximately 6-fold in the first 9 days after damage, and no macroscopic ulcer was observed 32 days after damage (Figure 2A). *H. pylori* had no effect on initial damage size at day 3 (1 Day after inoculum), however *H. pylori* significantly delayed ulcer healing such that at day 32 the ulcers were still prominent (Figure 2A).

### Preferential colonization of ulcerated tissue by *H. pylori*

In the same experiments, we compared the time course of bacterial colonization in the ulcerated tissue *versus* the contralateral region of undamaged tissue on the opposite side of the same stomach, using small tissue regions collected as shown in Figure 1D. We used two independent methods to measure *H. pylori* colonization of tissue: bacterial culture to obtain CFU (Figure 2B) and PCR of the *H. pylori* ssa gene (Figure S3) [25]. Control experiments confirmed the accuracy of the *ssa* PCR method *versus* CFU determination (Figure S3). Both methods identified significantly higher *H. pylori* colonization at the ulcer site *versus* the undamaged area of gastric tissue, at 1, 7 and 30 Days after inoculation (Figure 2B and S3). Both methods also reported undetectable levels of *H. pylori* in control (uninfected) tissues, confirming the specificity of our techniques. Additional control experiments examined conditions of no surgery and sham surgery (in the latter case applying PBS instead of acetic acid) to confirm that *H. pylori* colonization was not affected by these factors at either Day 1 or Day 7 after inoculum (Figure S4). Figure S4 also confirms that bacterial growth between Day 1–7 is not affected by surgery.

We also measured whether ulceration changed the total gastric bacterial load, and separately quantified gastric *Lactobacilli*. In mice not treated with *H. pylori*, PCR of 16S rRNA reported no difference in total bacterial load between the ulcers and healthy regions, suggesting that ulceration did not generally increase bacterial colonization (Figure 2C). As predicted, gavage with *H. pylori* increased total bacterial load *versus* the control condition in absence of both ulceration and *H. pylori* (Figure 2C). PCR for the genus *Lactobacillus*, a documented stomach commensal, confirmed the presence of the bacteria in control and *H. pylori* infected stomachs, but *Lactobacillus* did not preferentially colonize ulcerated tissues in either the absence or presence of *H. pylori* (Figure 2D). As reported by others, we observed that *Lactobacillus* increased at 30 Days after *H. pylori* inoculation [26]. Since this was not observed at earlier time points, this *H. pylori*-induced gastric microbiota change is a more long time effect. Combined, these results suggest that the observed preferential colonization of damaged tissue is specific to *H. pylori*.

**Figure 3:**
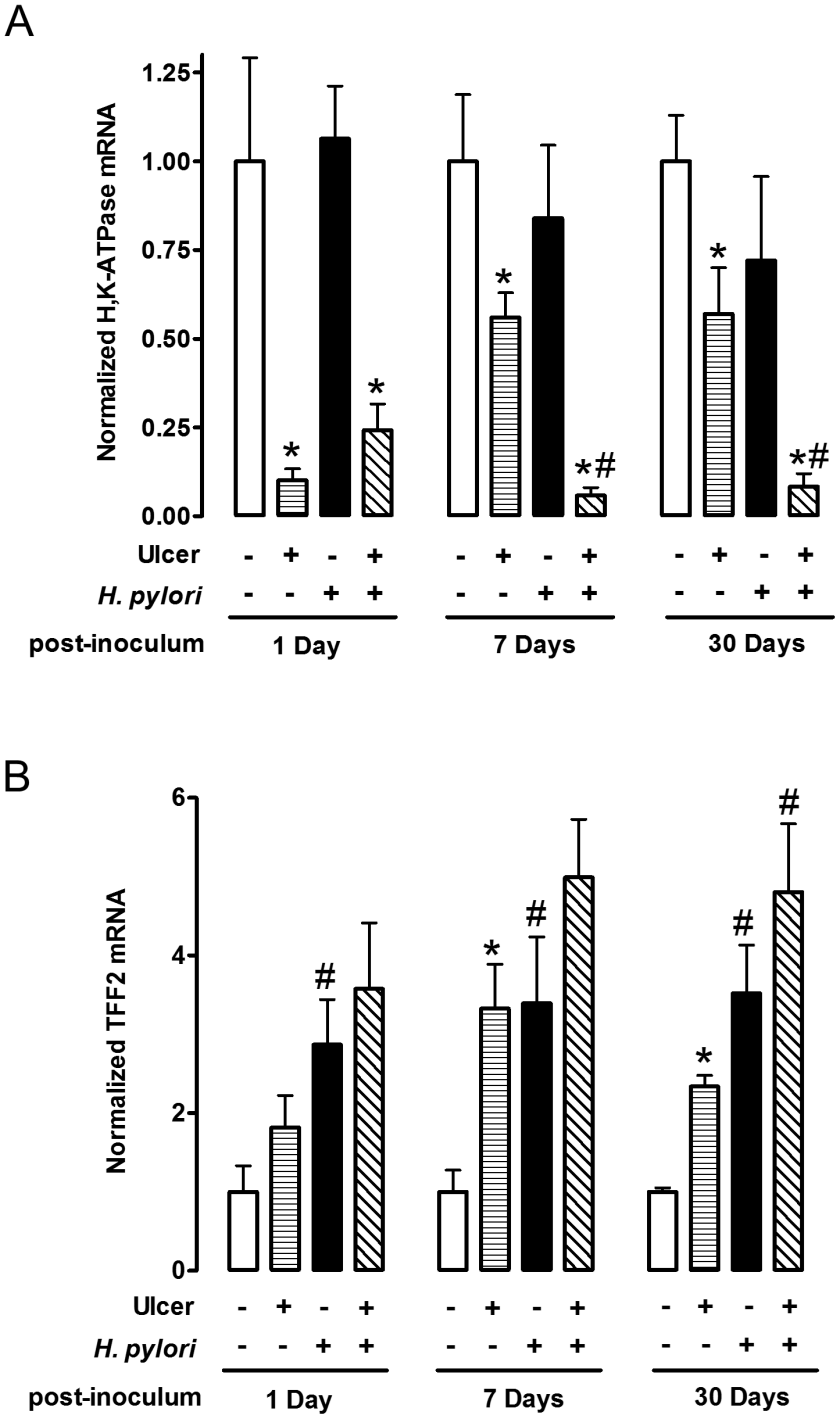
Effect of *H. pylori* on gene expression change during ulcer healing. Gastric ulcer was induced by topical serosal application of acetic acid. A single gavage of 10^6^ *H. pylori* was performed 2 days after ulcer induction. Ulcerated or non-ulcerated area were harvested 1, 7 or 30 Days after *H. pylori* inoculation. H,K-ATPase (A), and TFF2 (B) mRNA was detected by real-time PCR. Data are shown as fold change normalized to non-ulcerated region of uninfected group (ulcer −, *H. pylori −*). mean ± SEM. *, p<0.05 vs. intact region. ^#^, p<0.05 vs. no *H. pylori* inoculation group.

**Figure 4:**
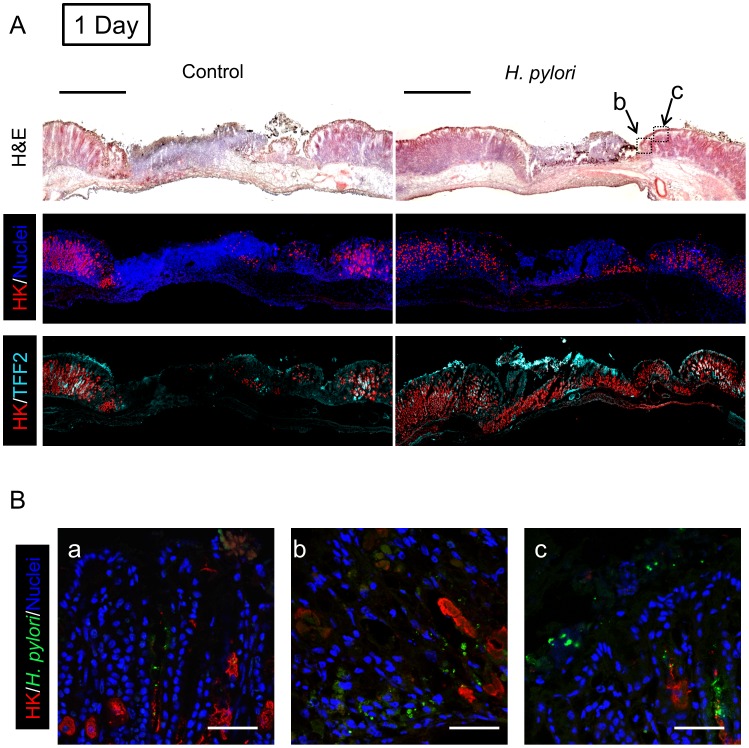
Morphology of gastric ulcerated tissue 1 day after *H. pylori* inoculation. Gastric ulcer was induced by topical serosal application of acetic acid. In some animals, a single gavage of 10^6^ SS1 *H. pylori* was performed 2 days after ulcer induction. Gastric tissue was isolated 3 days after ulcer induction (1 Day after *H. pylori* inoculum), and serial sections processed as described in Methods. Results are compared from the same tissue and sectioning series, although adjacent sections are not always presented. (A) Sections of uninfected (control) or *H. pylori* infected tissues are compared. Images show H&E staining, dual staining for H,K-ATPase (HK: red) and cell nuclei (blue), and dual stain for H,K-ATPase (HK: red) and TFF2 (cyan). Bar = 1 mm. (B) Tissue was also stained for *H. pylori*. Higher magnification of serial sections from (a) non-ulcerated corpus region of *H. pylori* infected stomach, or (b, c) around inset area (Z-focal plane ± 50 µm) indicated in A (H&E staining). Cell nuclei (blue), *H. pylori* (green), H,K-ATPase (HK: red). Bar = 50 µm.

**Figure 5:**
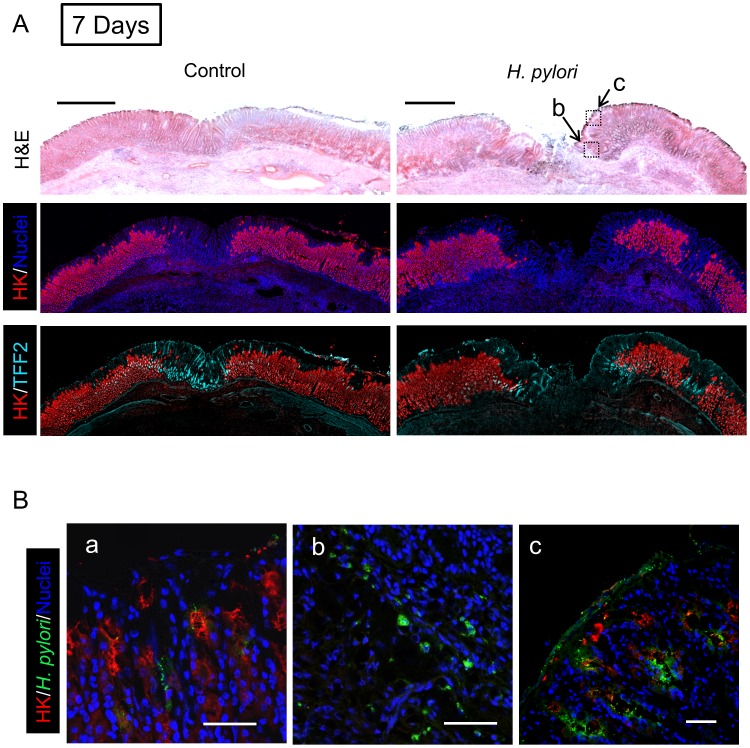
Morphology of gastric ulcerated tissue 7 days after *H. pylori* inoculation. Gastric tissue was isolated 9 days after ulcer induction (7 Days after *H. pylori* inoculum), and serial sections processed as described in Methods. (A) Sections of uninfected (control) or *H. pylori* infected tissues are compared as in Figure 4. Bar = 1 mm. (B) Tissue was also stained for *H. pylori*, and results presented as in Figure 4. Higher magnification of serial sections from (a) non-ulcerated corpus region of *H. pylori* infected stomach, or (b, c) around inset area indicated in A. Bar = 50 µm.

**Figure 6:**
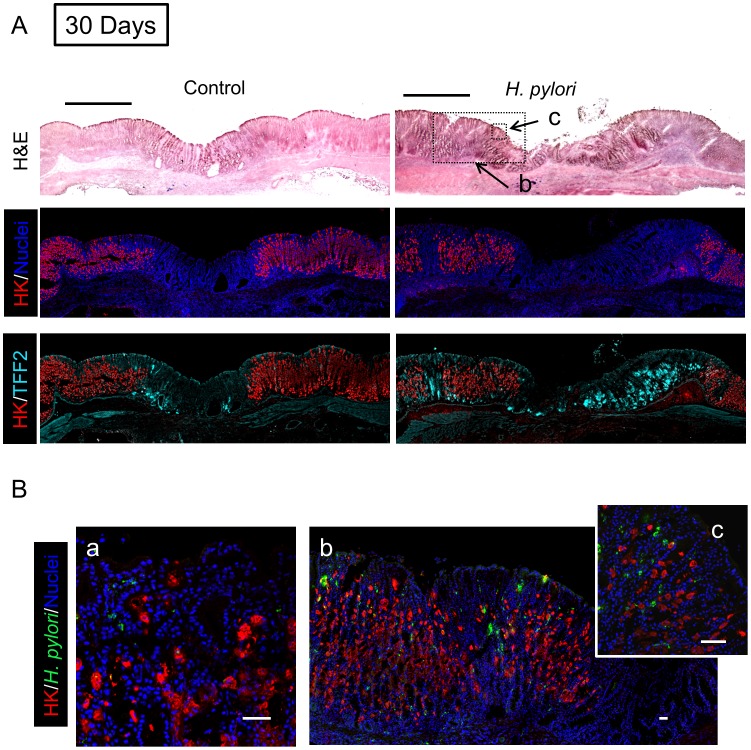
Morphology of gastric ulcerated tissue 30 days after *H. pylori* inoculation. Gastric tissue was isolated 32 days after ulcer induction (30 Days after *H. pylori* inoculum), and serial sections processed as described in Methods. (A) Sections of uninfected (control) or *H. pylori* infected tissues are compared as in Figure 4. Bar = 1 mm. (B) Tissue was also stained for *H. pylori*, and results presented as in Figure 4. Higher magnification of serial sections from (a) non-ulcerated corpus region of *H. pylori* infected stomach, or (b, c) around inset area indicated in A. Bar = 50 µm.

We examined two proteins by real-time PCR (Figure 3) and immunofluorescence (Figure 4–6) to interrogate tissue architecture and *H. pylori* tissue localization during the ulcer repair cycle. Trefoil factor 2 (TFF2) is a marker for gastric mucous cells, and a peptide that has been shown to promote repair of gastric damage [12]. H,K-ATPase mediates the gastric acid secretion that can oppose ulcer healing [24] and is a classic marker of parietal cells. Three days after ulcer induction in the absence of *H. pylori*, H&E staining (Figure 4A) showed many necrotic or apoptotic cells present at the ulcerated area (the ulcer crater recognized by macroscopic imaging in Figure 1D), and both PCR of ulcerated tissue (Figure 3A) and immunostaining (Figure 4A) showed the same area depleted of parietal cells. At this time point, *H. pylori* inoculation (for 24 hrs; Day 1) did not change these features recognized by macroscopic imaging (Figure 1D), PCR (Figure 3A) or histology and immunostaining (Figure 4A). *H. pylori* did, however, increase the presence of TFF2 recognized by both PCR and immunostaining (Figures 3B and 4A). By immunostaining, *H. pylori* bacteria were identified at the tissue surface and within gastric gland lumens (Figure 4B), while we failed to detect *H. pylori* in non-*H. pylori* infected stomachs (Figure S5). In infected stomachs, *H. pylori* appeared most abundantly in the ulcerated tissue and at ulcer margins (Figure 4B,b–c), but were also identified in undamaged tissue (Figure 4Ba).

In tissue at Day 7 (Figure 5) and Day 30 (Figure 6) after *H. pylori* inoculation (or 9 and 32 days post-ulcer induction), we observed the gastric epithelium still displayed altered distributions of H,K-ATPase and TFF2. In the uninfected tissues at both of these later time points the ulcer was healed and the epithelium had fully regenerated, but both immunostaining and PCR reported a sustained decrease in parietal cells (H,K-ATPase) and increase of mucous cells (TFF2) in the area that had been damaged. A more extensive comparison of the stomach distribution of these two proteins across the corpus at day 9 (Figure S6) confirms that these alterations are restricted to the area of gastric damage. In the *H. pylori* infected tissues at these later time points, no ulcer crater was visible macroscopically 32 days after ulceration (Day 30 after *H. pylori* inoculum), but histologically the tissue showed partial epithelialization and inflamed tissue at ulcer margins (Figure 5A & 6A). In this condition, both PCR and immunostaining report that infected ulcerated tissue has reduced parietal cells (H,K-ATPase) compared to infected tissue from non-ulcerated areas. In contrast, PCR reports an elevation in TFF2 in both intact and ulcerated areas of the infected tissue suggesting a general effect of *H. pylori* infection on TFF2 mRNA abundance (Figure 3B). In contrast, although TFF2 immunostaining is generally elevated in the damaged and ulcer margin areas at these later time points, we could not discern a consistent increase in TFF2 protein levels in general non-ulcerated tissue (Figure 5 and 6). Differences between mRNA and protein levels are commonly observed, and in this case discrepancies may be compounded in the PCR analysis by the need to collect tissues with variable amounts of ulcerated tissue over this repair time course. *H. pylori* bacteria were identified at both tissue surface and within gastric gland lumens at Day 7 after inoculation, but only within glands at Day 30 (Figure 5B and 6B). Qualitatively, *H. pylori* density appeared highest at ulcer margins. These results suggest that *H. pylori* preferentially colonizes the ulcerated tissue at the early time point, followed by migration/expansion to surrounding viable epithelial cells as colonization is established.

### Role of motility and chemotaxis in ulcer colonization

**Figure 7:**
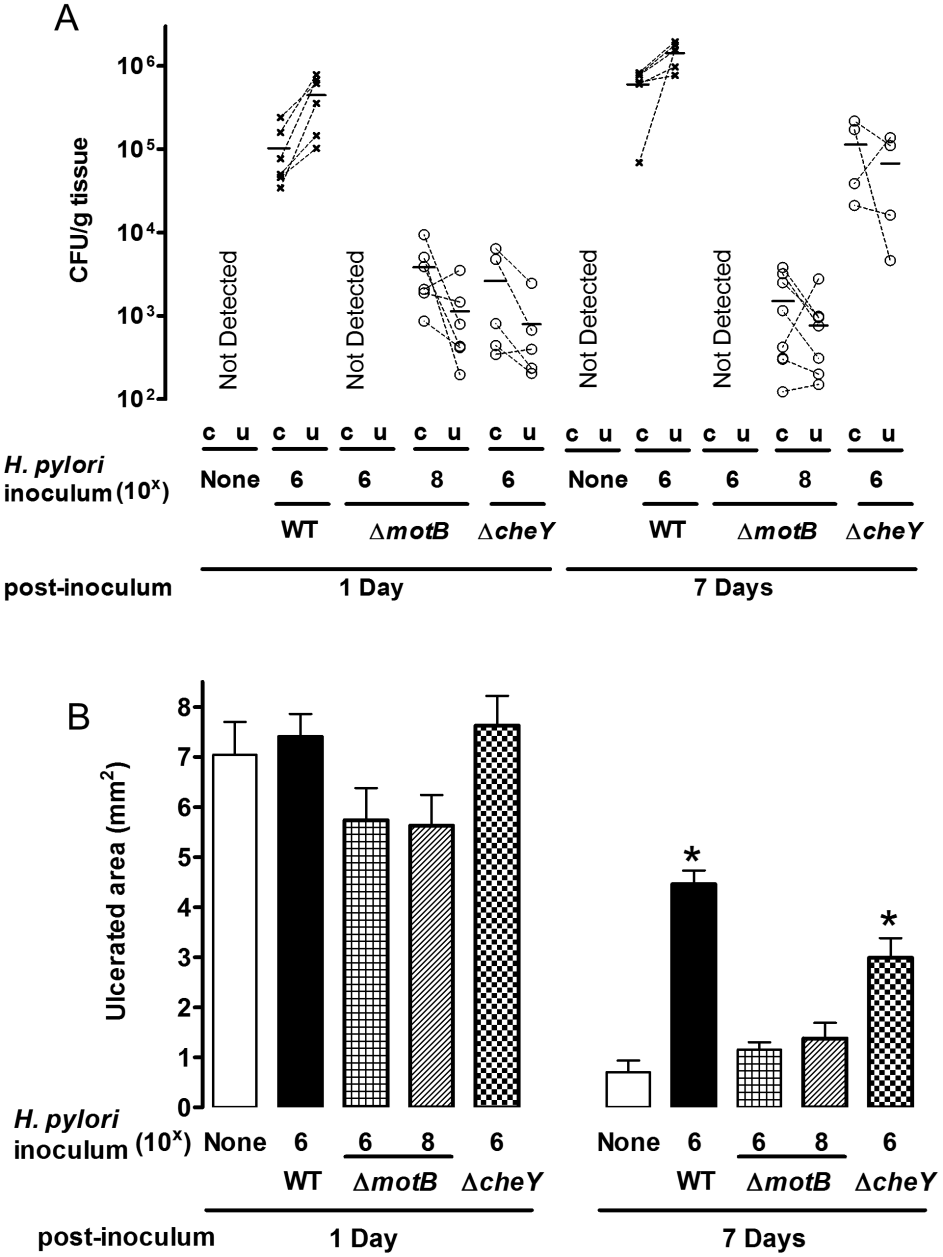
Effect of Δ*motB* and Δ*cheY* mutant *H. pylori* on gastric ulcer healing. Gastric ulcer was induced by topical serosal application of acetic acid. A single gavage of 10^6^ or 10^8^ Δ*motB* (n = 6), or 10^6^ Δ*cheY* (n = 4–6) *H. pylori* was performed 2 days after ulcer induction. Wild-type *H. pylori* data from Figure 2 are included for comparison. Ulcerated (u) or non-ulcerated control area (c) were harvested 1 or 7 Days after *H. pylori* inoculation. (A) Harvested gastric tissue was homogenized and *H. pylori* cultured on plates to obtain CFU/g tissue, with lines connecting tissue from the same animal to indicate trends. (B) Gastric ulcer size was measured. Mean ± SEM. *, p<0.05 vs. uninfected control.

To test the importance of *H. pylori* motility and chemotaxis in the preferential colonization of ulcerated tissue at early time points (1–7 Days after inoculation), we used *H. pylori* Δ*motB* or Δ*cheY* mutants. Prior to use, we confirmed the previously described motility phenotypes of these strains [2], [3]. As expected, Δ*motB* mutants are not motile, Δ*cheY* mutants are motile but do not change direction, and wild-type strains display normal motility and pH sensitivity (Figure S1 and S2). After inoculation with 10^6^ Δ*motB*, we failed to detect *H. pylori* colonization in the stomach (Figure 7A). However, Δ*motB* could colonize the stomach if we infected with a higher dose of 10^8^ (Figure 7A). Under this condition, we recovered ∼100 fold lower CFU/g of this mutant as compared to wild-type (Figure 7A). These findings are consistent with the documented elevated infectious dose and poor colonization properties previously reported for strains lacking motility [2]. Although there was moderate colonization by the Δ*motB* strain, it did not inhibit ulcer healing (Figure 7B) as compared to the wild-type. In addition, *H. pylori* Δ*motB* displayed no difference in the colonization of undamaged and ulcerated areas of stomach at either time point (Figure 7A). In contrast, 10^6^ Δ*cheY* colonized the stomach initially at low levels that increased over time (Figure 7A), and Δ*cheY* inhibited ulcer healing (Figure 7B). Results suggest Δ*cheY* grows reasonably in gastric tissue, consistent with previous findings [27]. In contrast to what we observed with wild-type *H. pylori*, Δ*cheY* colonized undamaged and ulcerated areas of stomach equally at either time point (Figure 7A). These results suggest that motility and chemotaxis facilitate the ability of *H. pylori* to colonize damaged gastric tissue to elevated levels.

**Figure 8:**
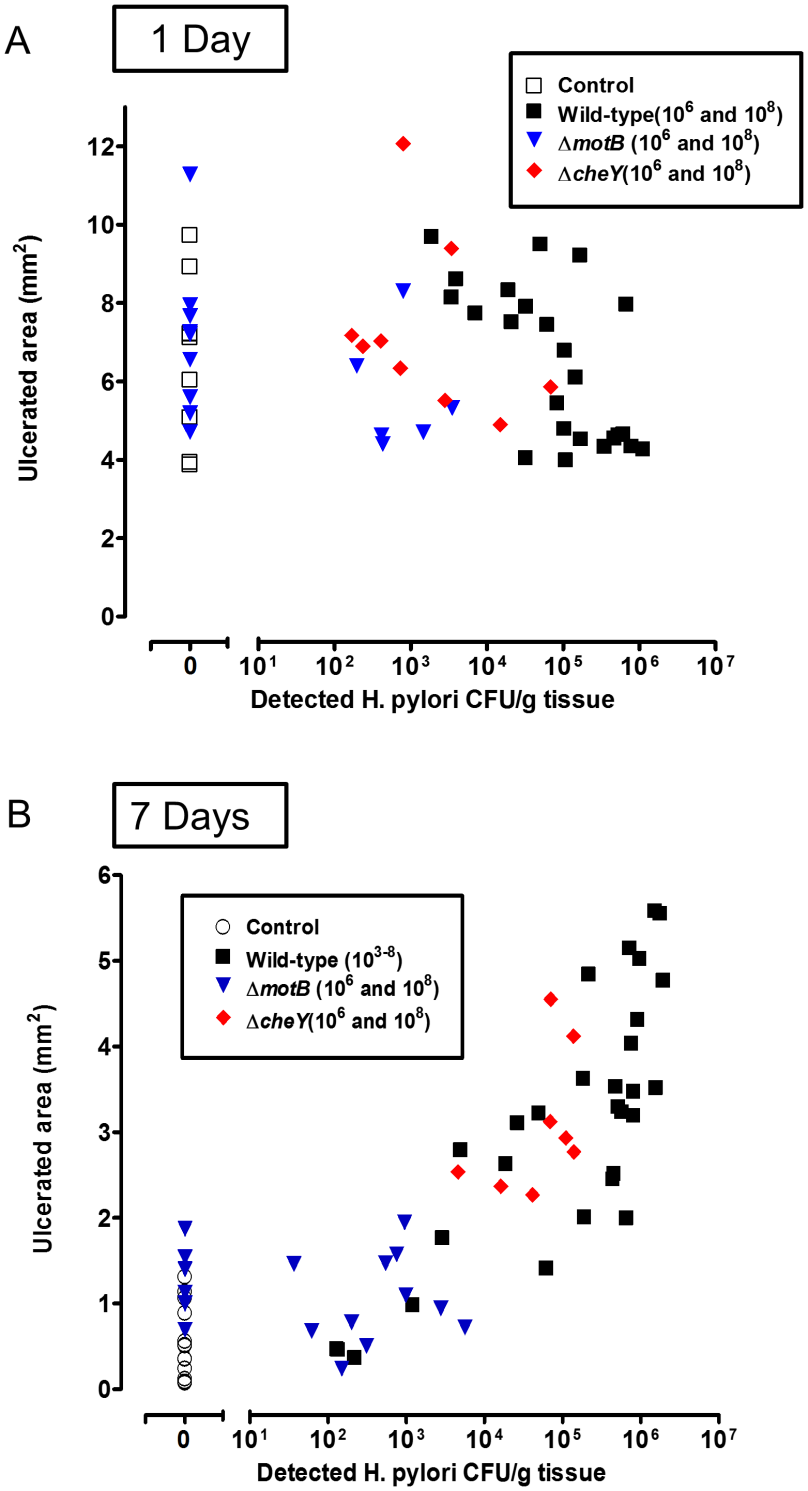
Relationship between ulcer size and *H. pylori* abundance. All data collected from all acetic acid ulceration experiments in this paper at (A) 1 Day or (B) 7 Days after *H. pylori* inoculation with the indicated strain. Results correlate the measured ulcer size vs *H. pylori* CFU/g tissue in the ulcerated area from the same mouse. Each data point is from a separate mouse.

In Figure 8, we plotted all data of wild-type and mutant *H. pylori* CFU/g in ulcerated regions *versus* the ulcer size from the same animal. As expected, 24 hr after inoculation, there is no correlation between ulcer size and *H. pylori* CFU (Figure 8A). In contrast, at Day 7 after inoculation (9 days after ulceration), we observed a strong correlation between bacterial load of all *H. pylori* genotypes and ulcer size (Figure 8B). This correlation suggests that bacterial load leads to compromised ulcer healing independent of *H. pylori* genotype. Once colonization is established, other virulence factors independent of chemotaxis and motility may slow ulcer repair. These results also suggest that chemosensing and motility help *H. pylori* target regions of tissue damage, and increase the efficiency of colonization.

### *H. pylori* chemotaxis immediately after detecting gastric damage

Our observations above show that *H. pylori* preferentially colonizes damaged tissue within one day after inoculation, but it was not clear when within this 24 hour period *H. pylori* localized with the damaged tissue, so we examined *H. pylori* behavior at earlier time points. Pilot experiments applied fluorescently tagged wild-type *H. pylori* directly to the surface of the surgically exposed gastric mucosa of anesthetized animals, whose stomachs were previously ulcerated by acetic acid. As shown in Figure S7, intravital confocal microscopy reported wild-type *H. pylori* were found more abundantly in ulcerated areas than in intact tissue within 1 hr of bacterial addition. In contrast, Δ*cheY* was less abundant in both intact and ulcerated tissue (Figure S7). This model was not pursued further since acetic acid ulcers were too large and heterogeneous to test in the same microscopic field if *H. pylori* selectively accumulates at a site of injury.

**Figure 9:**
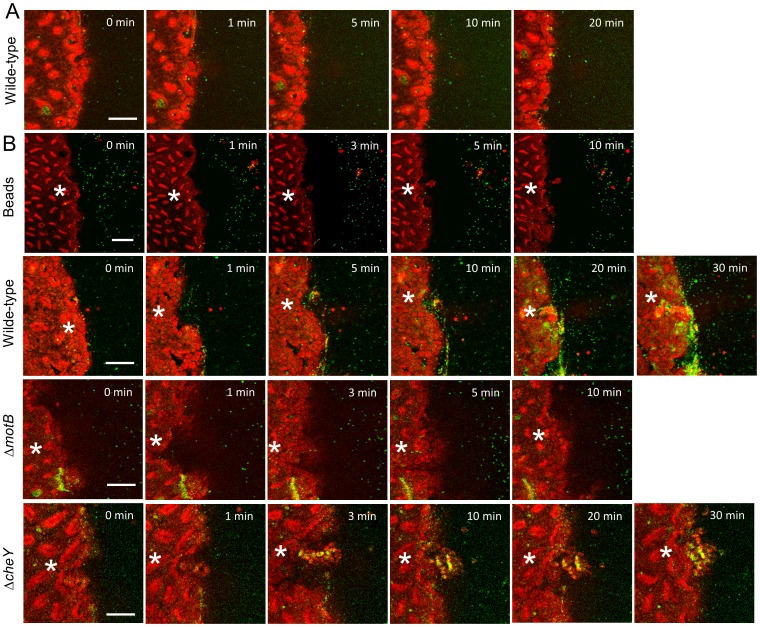
*H. pylori* accumulation near gastric surface after microscopic damage. Fluorescently labeled *H. pylori* or beads were added to luminal fluid bathing the exposed gastric mucosa. Two-photon damage was imposed on a microscopic region of the epithelium as described in Methods. Images show representative time course of confocal imaging of beads or *H. pylori* (green) in parallel with confocal reflectance (red), without (A) or with (B) photodamage (damage site indicated by *asterisk*) imposed directly after time zero. As indicated, each time series shows outcomes tracking labeled beads, *H. pylori* SSI (wild-type), Δ*motB*, or Δ*cheY*. Bar = 50 µm.

**Figure 10:**
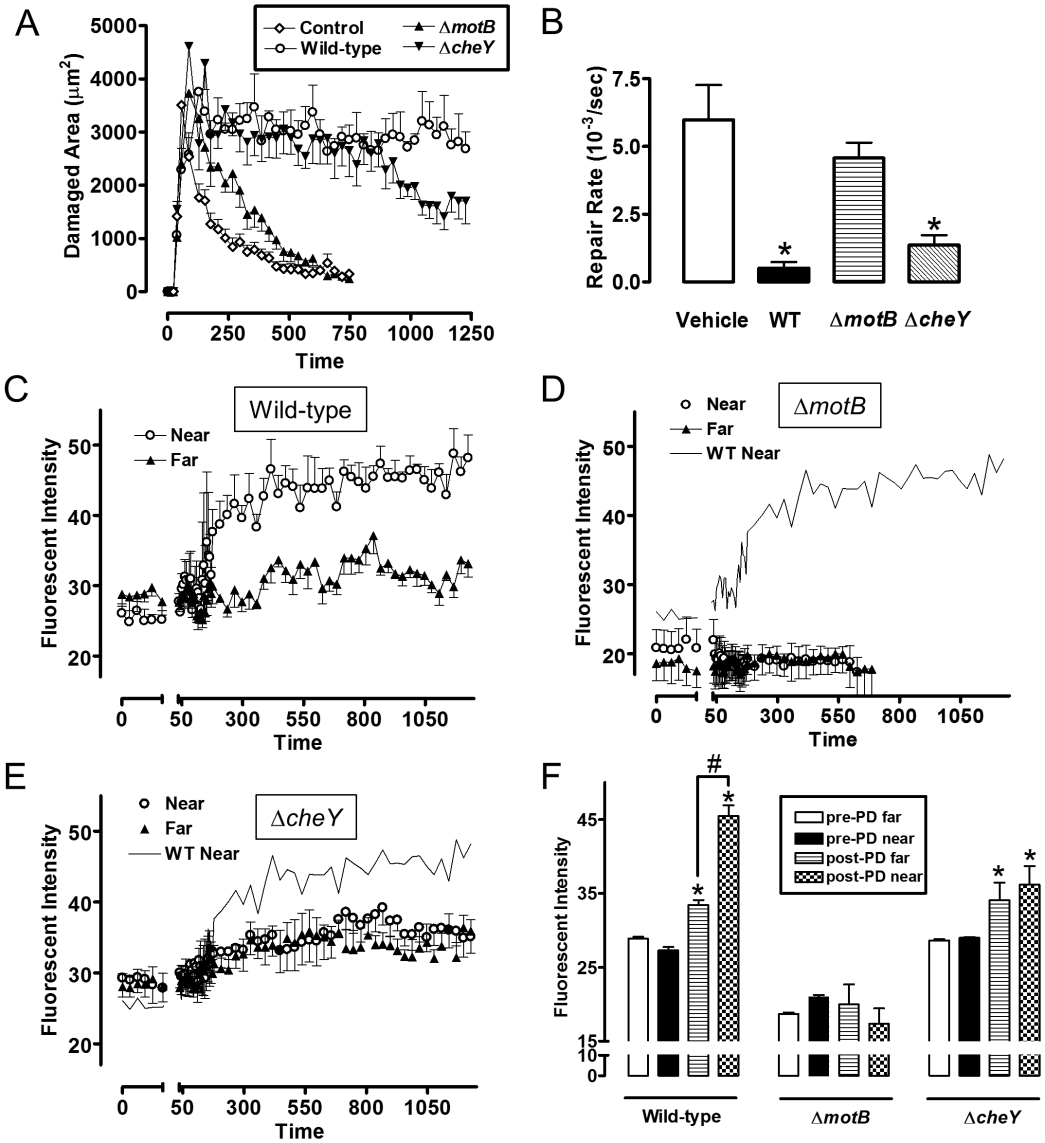
Quantifying effect of *H. pylori* on gastric epithelial repair after microscopic damage, and the localization of *H. pylori* during repair. Experiments were performed as in Figure 9, and results compiled. All results mean ± SEM. (A) Time course of the measured damage area, in the presence or absence of the indicated *H. pylori* strain. n = 5–8 damage/repair cycles (B) Rate of repair measured as described in Methods, in the presence or absence of the indicated *H. pylori* strain. *, p<0.05 vs vehicle. (C–E) measures of fluorescence intensity of the indicated *H. pylori* strains measured in the luminal space < 50 µm from the photodamage (PD) site (Near) or >100 µm from the damage site (Far). wild-type (n = 8), Δ*motB* (n = 6), Δ*cheY* (n = 6). (F) Analysis of results from panels C–E. Fluorescent intensity values measured directly prior to damage (pre) and 10 min after damage. *, p<0.05 vs same region pre-PD. #, p<0.05 near vs. far.

Therefore we used established two-photon microscopy methods to induce microscopic epithelial lesions in surgically exposed mouse gastric mucosa and then tracked repair in real-time over 15 min [11], [12], [28]. In initial experiments, we added fluorescent beads (1.0 µm diameter) to the gastric luminal superfusate, then induced photodamage of 3–5 gastric surface epithelial cells by two-photon laser. We then measured the rheological properties of the *in vivo* injury site environment. As shown in Figure 9B, S8 and Movie S2, fluorescent beads moved away from gastric tissue after damage, suggesting that the injury creates fluid flow away from the tissue into the lumen. Fluorescently labeled *H. pylori* Δ*motB* showed a similar passive flow away from the injury site in response to gastric damage (Figure 9B). In contrast, wild-type *H. pylori* rapidly accumulated at the two photon-induced damage site (Figure 9B) and this tissue accumulation was not observed in the absence of imposed damage (Figure 9A). *H. pylori* Δ*cheY* gradually accumulated at the gastric surface, but Δ*cheY* did not show an obvious affinity for the site of gastric damage. Results from such time course experiments were quantified by comparing surface intensity of *H. pylori* fluorescence at the site of damage *versus* surface regions (>50 µm) distant from damage (Figure 10C–E). Results from multiple experiments are compiled in Figure 10F, showing that Δ*cheY* had more limited surface accumulation than wild-type but greater than the strain lacking motility, and that only wild-type responded to damage with a preferential accumulation at damage sites.

In the same experiments evaluating bacterial surface accumulation in real time, we measured speed of gastric repair. Figure 10A shows the time course of repair, and Figure 10B shows the value of repair rate compiled from multiple experiments. Compared to the absence of bacteria (vehicle control), both wild-type and Δ*cheY* inhibited repair of damage, but Δ*motB* did not.

## Discussion

*H. pylori* chemotaxis genes have been identified as acid-induced genes and virulence factors that promote colonization initially and throughout infection, as well as promote *H. pylori* disease [4], [27], [29]. In *H. pylori*, however, the *in vivo* host-derived chemotactic factors regulating interactions of *H. pylori* with gastric tissue have been difficult to study. In this report, we used mouse models of ulceration and superficial epithelial damage to study the mechanisms employed by *H. pylori* to colonize the gastric niche. We demonstrate that *H. pylori* preferentially colonizes at an injury site compared to healthy regions of the stomach, with the highest efficiency of colonization requiring contributions of the chemotaxis system and flagellar motor activity in sensing the location of damaged tissue, and migrating towards the damage.

In our experiments, *H. pylori* can colonize gastric tissue in the absence of chemotaxis or even bacterial motility. Consistent with previous results [2], [3], our results suggest that high bacterial inocula can overcome the need for chemotaxis and motility. This finding is likely due to the fact that at supra-physiologic concentrations, there is increased probability that *H. pylori* will locate the correct niche by chance. Thus Che^−^ and Mot^−^ bacteria can establish colonization by luckily landing in favorable growth conditions and then engaging the mechanisms for attachment and other virulence factors normally engaged downstream of the chemotaxis functions.

Our study makes the striking observations that not only can *H. pylori* slow the repair of macroscopic ulcers over a period of 30 Days, but *H. pylori* in the gastric lumen can find damaged tissue and slow repair in a matter of minutes. Many investigators have confirmed that *H. pylori* inhibits ulcer healing induced by acetic acid [30]–[32], and one interesting study showed that gastric ulcer relapse occurs rapidly following *H. pylori* infection [30]. Using Mongolian gerbils, these investigators demonstrated that 8 weeks post-ulcer induction, ulcers were healed. However inoculation with *H. pylori* at this time point lead to re-expression of gastric ulcer more rapidly compared to previously uninjured animals [30]. Although the *H. pylori* abundance at the ulcerated area was not specifically determined in this prior study, it can be speculated that *H. pylori* were able to sense damaged tissue and preferentially colonize this site for rapid re-ulceration. Results from our micro-lesion model do not yet allow us to determine what *H. pylori* factors slow gastric repair. However, in both the ulcer and micro-lesion models, the ability of the Δ*cheY* mutant to slow repair makes it clear that the chemosensing system is not necessary to compromise epithelial repair.

We observed a striking decrease in H,K-ATPase expression during gastric ulcer healing, consistent with previous reports [33], [34]. We extended these findings by demonstrating that H,K-ATPase expression remains suppressed in the regenerating epithelium even by 32 days after ulceration, similar to observations by Blom's group that gastric wound repair resulted in full re-epithelialization by one month but parietal cells only re-appeared at 3 months and the number of parietal cells in the healed gastric mucosa never reached pre-ulceration levels [33]. Since the regenerated epithelium is slow to restore a normal cell census of parietal and TFF2-containing mucous cells, it is clear that ulcer healing results in an altered gastric epithelium that will alter both local gastric function and sensitivity to further insult. Results suggest that *H. pylori* may further delay the restoration of a normal epithelium in part by further delaying the epithelial repair process.

It is not yet known what signals drive *H. pylori* toward the damaged tissue. Changes in luminal pH in the microenvironment near the gastric surface may be a signal used by *H. pylori* chemotaxis to colonize this niche. We have shown that the pH increases in the luminal juxtamucosal space near the microlesions induced by two-photon laser damage [11], [12]. Others have used pH electrodes to show pH increases over sites of pervasive superficial gastric damage [13]. In preliminary studies with a pH 3 luminal perfusate, we observed using fluorescent pH indicators that luminal pH adjacent to the ulcer tissue was significantly elevated compared with intact tissue (Figure S9). *H. pylori* has a well-established pH-tactic response. It is reported that most *H. pylori* are free-swimming in the mucus gel layer within ∼30 µm of the epithelial surface [7], [35], [36]. *H. pylori* furthermore are spatially oriented within the mucus layer based on pH gradients [7]. Disruption of these gradients, by equalizing luminal and surface pH, causes *H. pylori* to lose their direction of motion, and spread over the entire mucus layer [37]. Specifically, *H. pylori* are repelled by acidic regions *in vitro*, and this behavior is eliminated by deletion of one of the chemotaxis receptor genes, *tlpB*, as well as the general chemotaxis pathway [8], [15]. In this study, we report that Δ*cheY* behavior is also different from wild-type *H. pylori* in a low pH environment. Thus one might predict that *H. pylori's* response to migrate away from acidic conditions would drive it toward the more neutral conditions overlying the damaged tissue.

Hydrogen ion concentration is only one of numerous gradients that could be act as chemorepellents or chemoattractants after gastric damage. Our 2-photon experiments determined that *H. pylori* responds to injury within minutes, and therefore it is unlikely that any signals result from inflammatory products. Instead, they are likely to arise by release promptly from epithelial damage. There are many potential candidates for *H. pylori* chemoattractants, including pH (CO_2_, HCO_3_^−^, H^+^), serum factors, urea, and ions (Ca, Mg, Fe, Zn etc) [4], although their presence at the injury site is not yet known. Several of these are reported chemoattractants for *H. pylori in vitro*, but not yet established *in vivo* [4], [20]. Based on the ability to rapidly evaluate *H. pylori* accumulation at the site of injury, further studies can now determine the chemoattractant(s) in the injury environment. Since this accumulation also slows gastric repair within minutes, future work also offers an opportunity to explore the mechanism and genes allowing *H. pylori* to initiate its earliest pathophysiological effects.

Several pathogens including *H. pylori* require chemotaxis for colonization, but to our knowledge, this report is the first to show bacterial chemotaxis aiding colonization of damaged tissue. However, Amieva's group has shown that in the intestine, *Listeria monocytogenes* preferentially adheres at sites of epithelial cell shedding [38]; and this may be considered a physiologic correlate of the microscopic epithelial discontinuity that we have produced experimentally. Supporting this conjecture, the same group has also shown that *H. pylori* can form microcolonies over intercellular junctions of cultured non-gastric cells [39]. Combined, the evidence suggests that compromises to the epithelial barrier can be an invitation to bacteria. There are several examples of chemotaxis directing pathogens to specific niches *in vivo*. For example, *H. pylori* requires chemotaxis for optimal initial colonization of the corpus and antrum, but after months of infection chemotaxis is only required to promote stable colonization and bacterial proliferation in the gastric glands of the antrum [3], [27]. In contrast, *Vibrio cholerae* uses chemotaxis to restrict its colonization to the jejunum and ileum. Che^−^ *V. cholerae* are found in elevated numbers compared to wild type throughout the gastrointestinal tract [40]. As documented in this work, bacteria are exquisite biosensors of their environment. As we continue to study the role of chemotaxis *in vivo*, we will learn about the variety and importance of these niches for promoting bacterial colonization and disease pathogenesis.

## Materials and Methods

### Ethics statement

All mice experiments were conducted according to both Animal Welfare Act Regulations and Public Health Service Policy on Humane Care and Use of Laboratory Animals. Mice were maintained in an AAALAC approved facility and all animal studies followed protocol 04-03-08-01 that was approved by the Institutional Animal Care and Use Committee of the University of Cincinnati (Cincinnati, OH).

### Animal husbandry and surgery

Experiments used C57BL/6J mice (Jackson lab, Bar Harbor, ME). Animals were used for experiments at 3–6 month of age, were fed a standard rodent chow diet, and had free access to water.

Gastric ulcers were produced by acetic acid, according to a previously described method with slight modification [24]. In brief, under isoflurane anesthesia, the abdomen was incised and the intact stomach exposed. A microcapillary tube (0.7 mm in diameter: Drummond Scientific Co. Broomall, PA) filled with acetic acid (99%) was placed in contact with the exterior surface of the stomach corpus region and left in place for 25 sec. Buprenorphine hydrochloride (0.75 mg/kg i.p., Buprenex, Rechkitt Benckiser Pharmaceuticals Inc., Richmond, VA) was given as preemptive analgesia. After the acid was removed, the treated exterior of the stomach was wiped with gauze, the abdomen was closed, and the animals were routinely maintained with food and tap water. Using this procedure, no incision was made to the stomach as part of ulcer induction.

To examine ulcer healing, animals were sacrificed on day 3, 9, or 32 after ulceration, and the stomach was removed and opened along the greater curvature. The area (mm^2^) of ulceration was measured by digital caliper (Mitutoyo, Kanagawa, Japan). Since deep, well-defined ulcers were consistently observed 2 days following acid application [24], [41], *H. pylori* were gavaged at day 2 after the ulceration. Control uninfected animals received Brucella broth vehicle.

The surgical preparation of animals for *in vivo* gastric microscopic damage study has been described [11], [12], [42]. Briefly, mice were anesthetized with inactin (10 mg/kg i.p., Sigma, St. Louis, MO) and ketamine (50 mg/kg i.p., Phoenix, St. Joseph, MO, USA), then the stomachs of anesthetized mice were exteriorized and everted to expose the gastric mucosa. The mouse was placed in prone position such that a portion of the exposed mucosa protruded into a perfusion chamber on the stage of an inverted confocal/two-photon microscope (Zeiss LSM 510 NLO; Carl Zeiss, Jena, Germany), with the microscope stage enclosed and heated to keep the animal's body temperature at ∼37°C. The mucosal surface was exposed to fluorescently labeled *H. pylori*.

### Preparation and inoculation of *H. pylori*

*H. pylori* strain SS1 and isogenic mutants [2]–[4] were grown on Columbia blood agar plates (Remel, Lenexa, KS, USA) containing 5% defibrinated horse blood (Colorado Serum, Denver, CO, USA), 0.2% β-cyclodextrin (Sigma), 50 µg/ml cyclohexamide (Sigma), 5 µg/ml vancomycin (Sigma), 10 µg/ml trimethoprim (Sigma) and 5 µg/ml chloramphenicol (only for Δ*cheY* mutant, Sigma) for 4 days. Colonies from these plates tested positive for urease (BD Diagnostic Systems, Franklin Lakes, NJ, USA), catalase (using 3% H_2_O_2_) and oxidase (DrySlide, BD Diagnostic Systems). Bacteria harvested from the plate were grown in Brucella broth (BD Diagnostic Systems) supplemented with 10% fetal bovine serum and CO_2_ gas pack (BD Diagnostic Systems) in a humidified microaerophilic chamber (BBL Gas System, with CampyPak Plus packs, BD Microbiology, Sparks, MD, USA) in an incubator at 37°C for 16 to 18 hr without shaking. Bacteria were collected by centrifuge at 2000 rpm for 5 min, and resuspended in Brucella broth without serum. *H. pylori* were diluted in 50% glycerol (1∶100 dilution) and counted in haemocytometer. Each mouse received 200 µl Brucella broth containing 10^3^ to 10^8^ *H. pylori* bacteria. Control mice (uninfected group) received 200 µl of Brucella broth alone.

For *in vivo* gastric microscopic damage study, *H. pylori* was labeled with one of the following fluorescent dyes (Invitrogen, Carlsbad, CA, USA): cell tracker green CMFDA, cell tracker orange CMRA or 5-(and -6)carboxyfluorescein diacetate, succinimidyl ester (5(6)- CFDA, SE: CFSE)-mixed isomers. Briefly, fluorescent dye (5 µM) was added into Brucella broth containing *H. pylori* for 15 min at 37°C, then washed with Brucella broth twice using 0.22 µm filter. *H. pylori* were adjusted to 10^8^/ml for usage.

### Quantification of levels of *H. pylori* in mouse stomach tissue

The wet weight was measured of small regions of mouse gastric tissue collected from intact (right side of gastric corpus in Figure 1D) and ulcerated (left side of gastric corpus in Figure 1D) tissue. Tissue was homogenized by Tissue Tearor (Model: 985370-395, BioSpec Products) in 1 ml saline and 1–10 dilutions were spread on a Columbia blood agar plate containing 5% defibrinated horse blood, 50 µg/ml cyclohexamide, 5 µg/ml vancomycin, 10 µg/ml trimethoprim (and 5 µg/ml chloramphenicol only for CheY mutant). Plates were incubated for 5–7 days at 37°C in a humidified microaerophilic chamber with CO_2_ gas pack. Colonies were counted and data normalized using the tissue weight and expressed in CFU/g tissue.

### Quantification of total bacteria using real-time PCR

Quantitative analysis of bacterial load was performed by qRT-PCR using specific primers targeting different gut bacterial groups. Total RNA was isolated from either the intact or ulcerated area of stomach using TRI Reagent (Molecular Research Center) and single-stranded cDNA was synthesized by iScript cDNA synthesis kit (Bio-Rad, Hercules, CA, USA). Quantitative real-time PCR was performed using SYBR Green PCR Master Mix (Applied Biosystems) with following specific primers: 16S rRNA (Forward: 5′-ACTCCTACGGGAGGCAGCAGT-3′ and Reverse: 5′- ATTACCGCGGCTGCTGGC-3′) [43], Lactobacillus (Forward: 5′-GGAAACAGATGCTAATACCG-3′ and Reverse: 5′-CACCGCTACACATGGAG-3′) [44], SSA specific to *H. pylori* (Forward: 5′-TGGCGTGCTCTATTGACAGCGAGC-3′ and Reverse: 5′-CCTGCTGGGCATACTTCACCATG-3′), H,K-ATPase (Forward: 5′-AGATGGTGTTTGCTCGAACC-3′ and Reverse: 5′-TCCAGCAAGATCATGTCAGC-3′), TFF2 (Forward: 5′-GCAGTGCTTTGATCTTGGATGC-3′ and Reverse: 5′-TCAGGTTGGAAAAGCAGCAGTT-3′), or GAPDH (Forward: 5′-AACGACCCCTTCATTGAC-3′ and Reverse: 5′-TCCACGACATACTCAGCAC-3′). Each PCR amplification was performed in duplicate wells in a StepOnePlus Real-Time PCR System (Applied Biosystems).

### Histological assessment

Mouse intact or ulcerated stomach tissue was embedded in OCT and frozen and stored at −80°C. Serial cryosections (10 µm) were prepared by cryostat. The section was stained with hematoxylin & eosin to localize and evaluate damage severity. The adjacent sections including damaged area were then stained using anitbodies against *H. pylori*, TFF2 or H^+^/K^+^-ATPase. For immunostaining, sections were fixed with 4% paraformaldehyde followed by incubated with goat serum (4%) for 60 min. Sections were then incubated with primary antibodies indicated below for 60 min in room temperature. Primary antibodies to *H. pylori* (Rabbit polyclonal 1∶ 1000, Abcam) and H^+^/K^+^-ATPase alpha subunit (Mouse monoclonal 1∶ 1000, Thermo Science) or TFF2 (Rabbit polyclonal, 1∶200) were used. Secondary antibodies (Alexa 633-labeled goat anti-mouse IgGγ1: 1∶1000, and Alexa 488-labeled goat anti-rabbit IgG, 1∶1000 for *H. pylori*, 1∶ 400 for TFF2, Invitrogen, Carlsbad, CA, USA) were incubated for 60 min in room temperature. Nuclear DNA staining was performed by incubation with Hoechst 33342 (Invitrogen) at 1 µg/ml for 1 min.

### Live *H. pylori* quantitative imaging

*H. pylori* labeled with CMFDA or CFDA-SE (Invitrogen) fluorescence was imaged at 500–530 nm in response to 488 nm excitation, while CMRA was imaged at 565–615 nm in response to 560 nm excitation. *H. pylori* was loaded into uncoated μ-Slide chamber (ibidi) and monitored on the inverted fast scan confocal microscope (Zeiss LSM 7 LIVE; Carl Zeiss), with the microscope stage enclosed and heated at ∼37°C. To analyze bacterial swimming, the program Image J with manual tracking plugin was used. The number of stopping was counted from 4 sec motility traces and data showed as number of stopping per second.

### Gastric microscopic damage model

The method of inducing, tracking, and quantifying microscopic photodamage in the gastric surface epithelium has been described previously [11], [12], [28], [42]. Two-photon imaging of tissue NAD(P)H autofluorescence (Ti-Sa excitation 730 nm, emission 435–485 nm) was collected simultaneously with a confocal reflectance image (reflecting 730 nm light to show cell/tissue structure). In some experiments, CMFDA-labeled *H. pylori* was applied to chamber and monitored in parallel (Ex: 488 nm, Em: 500–550 nm). After collecting a set of control images using minimal laser power, a small rectangle region (≈200 µm^2^) of gastric surface epithelium in the corpus was repetitively scanned at high Ti-Sa laser power (350 mW average) for 150 iterations (requiring 5–15 sec). Photobleaching of endogenous NAD(P)H induced epithelial cell death and extrusion followed normally by epithelial restitution via epithelial cell migration in ∼15 min, as recorded by two-photon/confocal time course imaging.

Damage (and repair) was quantified from the time course of images as described [12], [28] using Metamorph software (ver. 6.3, Molecular Devices, Downington, PA, USA). Briefly, the damaged area was measured as the region of cellular loss of autofluorescence NAD(P)H, using confocal reflectance images to positively identify the location of cellular structures. In each experiment, we determined the time point displaying maximal damage area and estimated rates of epithelial restitution starting from this time with a single exponential curve fit to the changing size of damaged area over time [12]. Best fit values of the rate constant (κ) were used as estimates of the rate of restitution (estimate of the fractional recovery per time, in units of sec^−1^). In some experiments, intensity of *H. pylori* fluorescence at the gastric surface was measured. The damage-repair cycle was measured independently several times per animal in different locations of the corpus, and outcomes from at least 4 animals were compiled for each experimental protocol.

### Statistical analysis

All values are reported from representative experiments as the mean ± standard error of the mean (SEM) from multiple experiments. The number of repetitions is reported as the number of independent damage-repair cycles (n) analyzed, and the number of different animals used is also indicated. All results were reproduced in at least 4 animals. Statistical significance was determined using unpaired Student's T-test, or one-way ANOVA with Dunnett's multiple comparison post-hoc test. A p value of <0.05 was considered significant.

## Supporting Information

Figure S1 **Motility patterns of** ***H. pylori*** **wild-type and mutants at different pH.** Wild-type (SS1), Δ*motB*, and Δ*cheY H. pylori* were grown and fluorescently labeled as described in Methods, suspended in Brucella broth, placed in Ibidi chambers, and individual bacterial motion patterns recorded by confocal microscopy. Bacterial mutants with a defective flagellar motor (Δ*motB*) had normal rod shape, and were positive for urease, catalase and oxidase (data not shown). (A) Two dimensional motility tracking over 4 sec of *H. pylori* wild-type (n = 12–19), Δ*motB* (n = 10) or Δ*cheY* (n = 10–19) in Brucella Broth at the indicated pH. All motility tracks initiate at x,y = 0,0 and the indicated distance values are µm. (B) Compiled results of average velocity over 4 sec of indicated *H. pylori* genotypes, calculated from A.
(TIF)

Figure S2 **Variation of velocity over time of** ***H. pylori*** **strains at different pH.** Individual *H. pylori* motility measured as in Figure 1. (A) *H. pylori* SS1 wild-type (n = 6), or ΔCheY (n = 6) motility tracking for 4 sec in Brucella Broth at indicated pH. (B) Ratio of maximum/minimum velocity calculated from each 4 sec motility trace in A. (C) Numbers of stopping behavior were counted from each motility traces in A over the entire 4 sec time course and data are shown as number of stopping per second. Mean ± SEM. *, p<0.05 versus SS1.
(TIF)

Figure S3 **Detection of** ***H. pylori*** **ssa gene by qPCR.** The same suspensions of *H. pylori* at variable amount were either plated and counted as CFU, or mRNA was extracted and subjected to real-time PCR amplification of the ssa gene. (A) Comparison of plate CFU *versus* ssa detection of different *H. pylori* (latter reported as Ct value from qPCR). PCR products run on agarose gel to confirm single amplicon product over wide range of input *H. pylori*. (B) Detection of *H. pylori* ssa gene on ulcerated or non-ulcerated control area were harvested at 1 (n = 6), 7 (n = 6) or 30 (n = 4) Days after *H. pylori* inoculation, and data are shown as fold change relative to the corresponding contralateral (ulcer negative) region in the presence of *H. pylori*. mean ± SEM. *, p<0.05 vs. ulcer negative region.
(TIF)

Figure S4 **Effect of surgery on** ***H. pylori*** **colonization in the stomach.** Some animals were examined without any intervention (surgery −, ulcer −), while other animals underwent sham surgery performed by applying PBS to the exterior of the surgically exposed stomach (surgery +). The final group of animals underwent surgery and the conventional application of acetic acid to the exterior of the stomach to induce gastric ulcer (ulcer +). PBS or acetic acid was always applied to the left side of the stomach (L), and the right side (R) was not exposed to any chemical. A single gavage of 1×10^6^ *H. pylori* was performed in all animals, 2 days post-surgery in the groups where surgery was performed. At 1 or 7 Days post inoculum, tissue was collected separately from both sides of the stomach as depicted in Figure 1C, homogenized, and *H. pylori* cultured to obtain CFU. Data are presented as CFU/g tissue, with lines connecting tissue from the same animal to indicate trends. Significant difference at p<0.05 *vs. tissue from right side of stomach.
(TIF)

Figure S5 **Reactivity of** ***H. pylori*** **antibody on tissue, in the absence of** ***H. pylori*** **gavage.** Gastric ulcer was induced by application of acetic acid as described in Methods, and tissue collected at the same days as indicated in Figures 4–6. (A) Tissue dually stained for H,K-ATPase (HK: red) and *H. pylori* (green). Bar = 1 mm. (B) Tissue triple stained for H,K-ATPase (HK: red), DNA (blue), and *H. pylori* (green). Images are higher magnification of tissue from (a) non-ulcerated corpus region of stomach, or (b, c) ulcer margin areas indicated in rectangles of A (albeit from a different section). Bar = 50 µm.
(TIF)

Figure S6 **Specific localization of parietal and mucous cell re-distribution caused by ulceration.** Gastric ulcer was induced by application of acetic acid as described in Methods, with tissue evaluated 9 days after ulceration. Stereoscopic image (top) taken from 9 days control in Figure 1D, and red rectangle indicates region imaged by confocal microscopy in lower image, evaluating TFF2 (blue) and H,K-ATPase (red) immunostaining.
(TIF)

Figure S7 **Rapid** ***H. pylori*** **accumulation in the ulcerated area.** Gastric ulcer was induced by topical serosal application of acetic acid. Under anesthesia, gastric mucosa was exposed, and placed in the chamber on confocal microscope as described in the Methods. Hoechst 33342 (red) was given intravenously at 5 mg/kg before imaging. Fluorescent labeled wild-type or Δ*cheY H. pylori* (green) were applied to the gastric mucosa. Confocal microscopy images were taken in the ulcer margin or the non-ulcerated (intact) gastric tissue within 1 hr of applying bacteria. Bar = 10 µm.
(TIF)

Figure S8 **Fluorescent bead movement in the gastric lumen after epithelial damage.** 10^5^ fluorescent beads were added to gastric luminal fluid. Two-photon damage was imposed on a microscopic region of the exposed gastric mucosa as described in Methods. Confocal images of time course after photodamage (PD) imposed at time zero. Gastric tissue confocal reflectance (red), bead fluorescence (green). Asterisk indicates site of PD, Image analysis by Imaris 7.6 software (Bitplane) of bead motion over time. Each line tracks an individual bead, pseudo-colored to show progression of distance from tissue over 3 min. Asterisk indicates tissue site of PD. Each line tracks an individual bead, pseudo-colored to show X position on the image window. Bar = 50 µm.
(TIF)

Figure S9 **Luminal pH change adjacent to the ulcerated stomach.** Gastric ulcer was induced by acetic acid as described in Methods. The mouse was anesthetized and gastric surface surgically exposed for confocal imaging 3 days after ulceration. Gastric lumen was perfused with pH 7 saline containing 20 µM BCECF. BCECF was alternately excited by 488 nm and 458 nm, with 500–550 nm emission. 488 nm/458 nm ratio was fitted to calibration curve to convert to extracellular pH value as described previously [42]. Representative images/analysis shows gastric luminal pH as a function of distance from tissue (position zero) comparing (A) non-ulcerated stomach (intact) area, or (B) ulcerated area.
(TIF)

Movie S1 **Fluorescent labeled** ***H. pylori*** **motility.** Fluorescent labeled *H. pylori* was loaded into uncoated μ-Slide chamber (ibidi) and monitored on the inverted fast scan confocal microscope (Zeiss LSM 7 LIVE), scan speed at 100 ms/flame.
(AVI)

Movie S2 **Fluorescent bead movement in the gastric lumen after epithelial damage.** 10^5^ fluorescent beads were added to gastric luminal fluid. Two-photon damage was imposed on a microscopic region of the exposed gastric mucosa. Bead motion was tracked by Imaris 7.6 software (Bitplane) for 3 min as shown in Figure S8. Each line tracks an individual bead, pseudo-colored to show X position on the image window. Frames from this movie are shown in Figure S8.
(AVI)
