# Supplementary figures and images for: Motility and Chemotaxis Mediate the Preferential Colonization of Gastric Injury Sites by Helicobacter pylori
Source: PLoS Pathog. 2014 Jul 17;10(7):e1004275. doi: 10.1371/journal.ppat.1004275 (PMC4102597; doi:10.1371/journal.ppat.1004275)

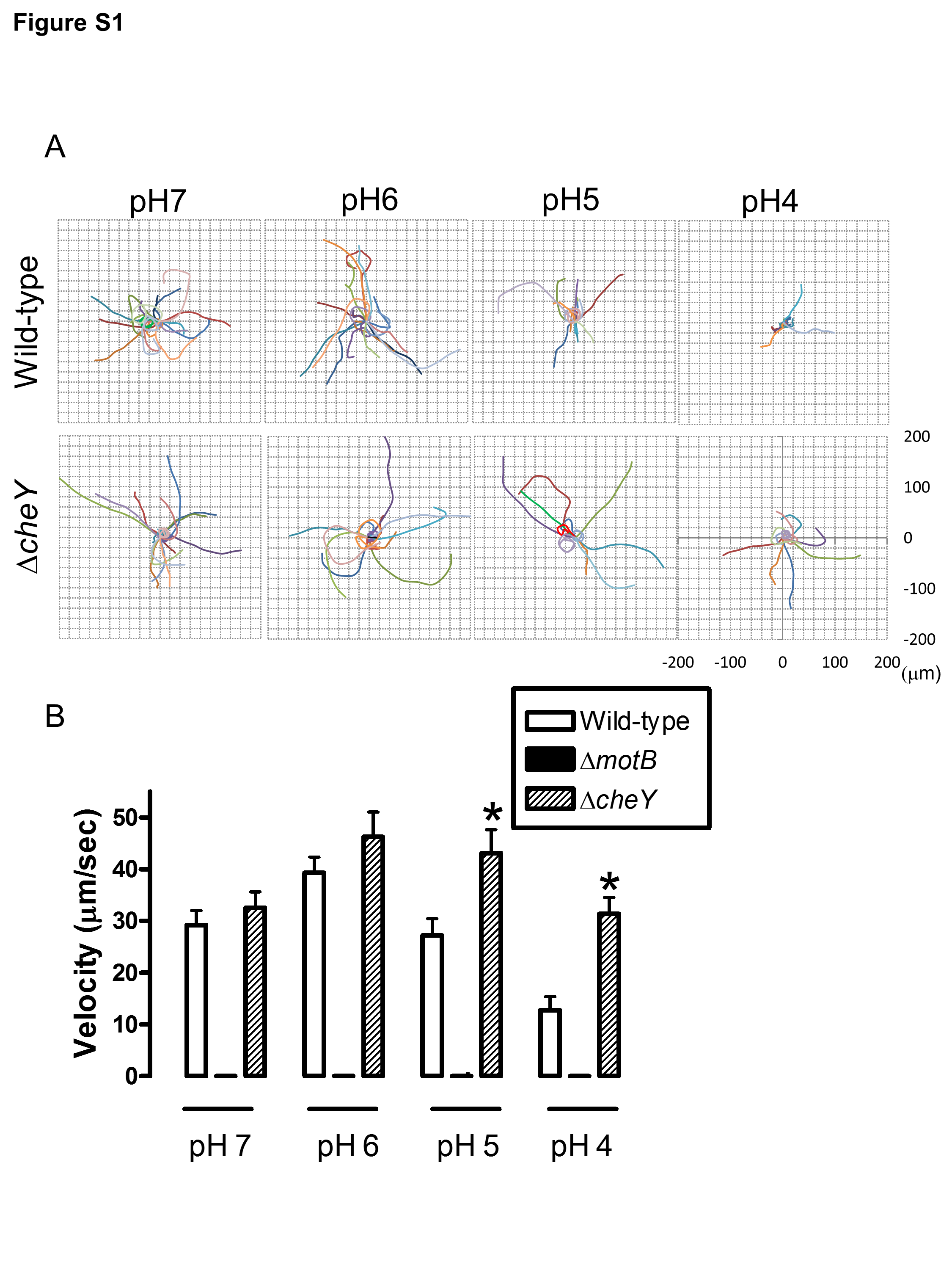

Supplement: Figure S1 — Motility patterns of H. pylori wild-type and mutants at different pH. Wild-type (SS1), ΔmotB, and ΔcheY H. pylori were grown and fluorescently labeled as described in Methods, suspended in Brucella broth, placed in Ibidi chambers, and individual bacterial motion patterns recorded by confocal microscopy. Bacterial mutants with a defective flagellar motor (ΔmotB) had normal rod shape, and were positive for urease, catalase and oxidase (data not shown). (A) Two dimensional motility tracking over 4 sec of H. pylori wild-type (n = 12–19), ΔmotB (n = 10) or ΔcheY (n = 10–19) in Brucella Broth at the indicated pH. All motility tracks initiate at x,y = 0,0 and the indicated distance values are µm. (B) Compiled results of average velocity over 4 sec of indicated H. pylori genotypes, calculated from A. (TIF) [file ppat.1004275.s001.tif]

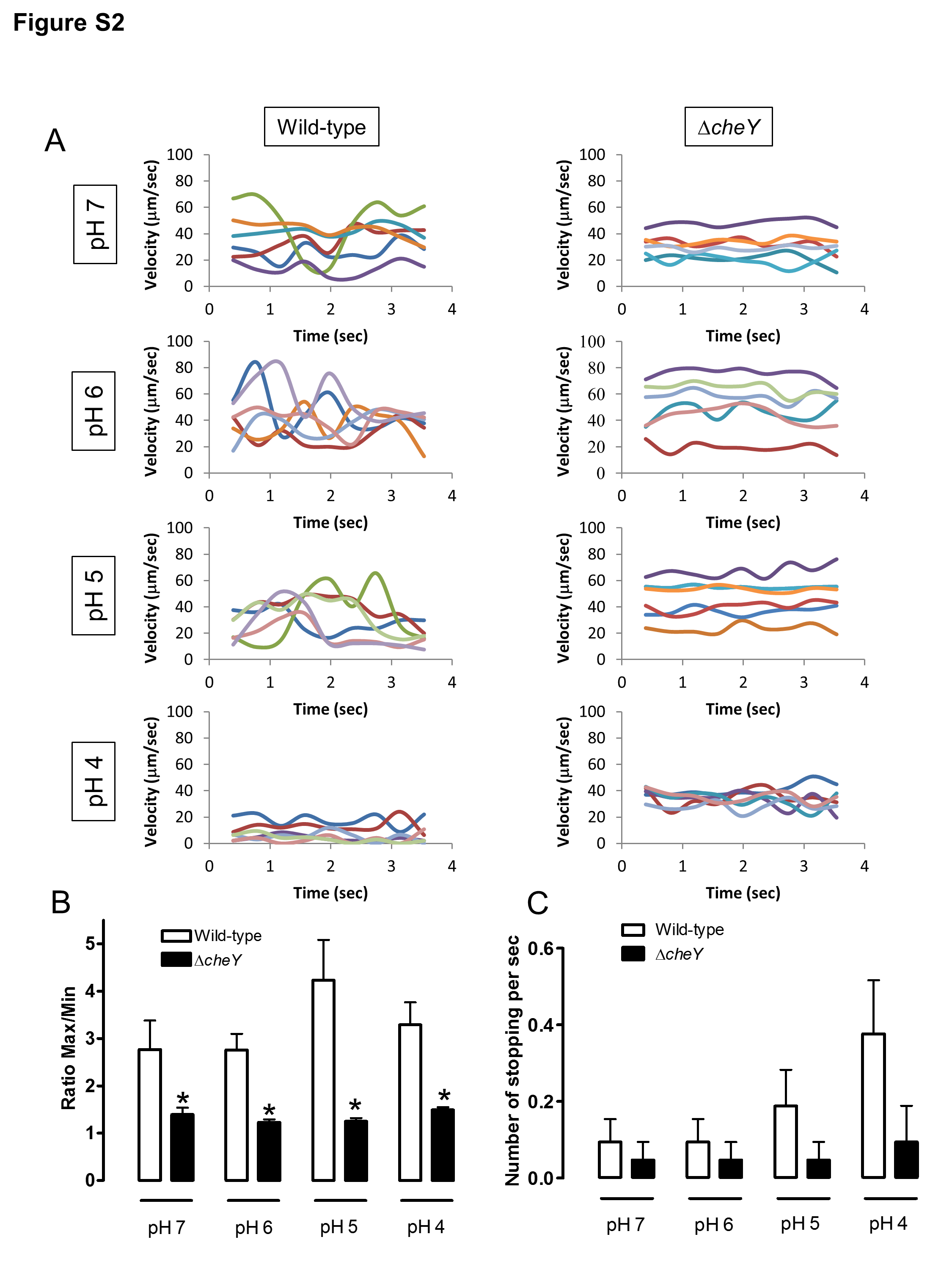

Supplement: Figure S2 — Variation of velocity over time of H. pylori strains at different pH. Individual H. pylori motility measured as in Figure 1. (A) H. pylori SS1 wild-type (n = 6), or ΔCheY (n = 6) motility tracking for 4 sec in Brucella Broth at indicated pH. (B) Ratio of maximum/minimum velocity calculated from each 4 sec motility trace in A. (C) Numbers of stopping behavior were counted from each motility traces in A over the entire 4 sec time course and data are shown as number of stopping per second. Mean ± SEM. *, p<0.05 versus SS1. (TIF) [file ppat.1004275.s002.tif]

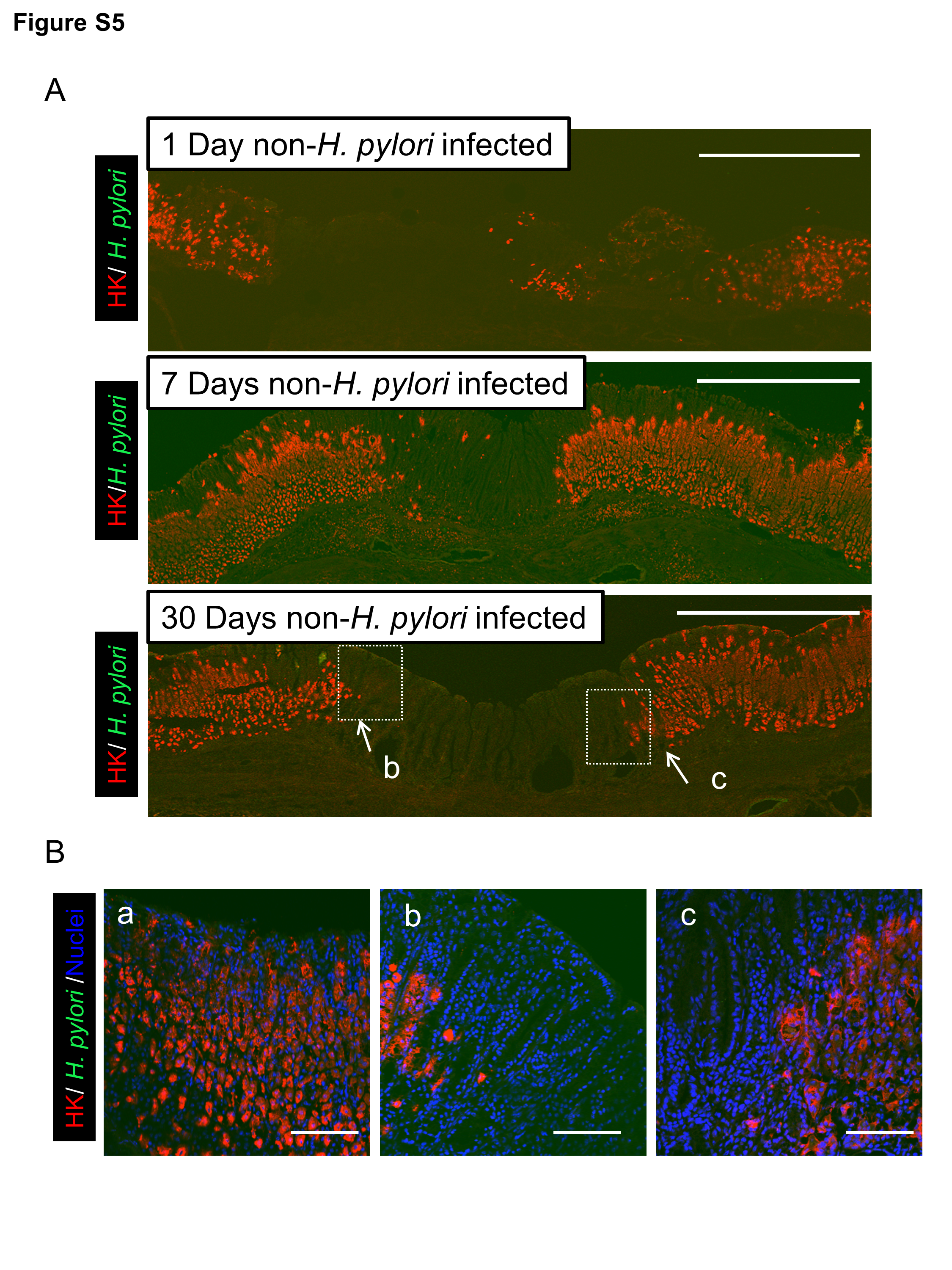

Supplement: Figure S5 — Reactivity of H. pylori antibody on tissue, in the absence of H. pylori gavage. Gastric ulcer was induced by application of acetic acid as described in Methods, and tissue collected at the same days as indicated in Figures 4–6. (A) Tissue dually stained for H,K-ATPase (HK: red) and H. pylori (green). Bar = 1 mm. (B) Tissue triple stained for H,K-ATPase (HK: red), DNA (blue), and H. pylori (green). Images are higher magnification of tissue from (a) non-ulcerated corpus region of stomach, or (b, c) ulcer margin areas indicated in rectangles of A (albeit from a different section). Bar = 50 µm. (TIF) [file ppat.1004275.s005.tif]

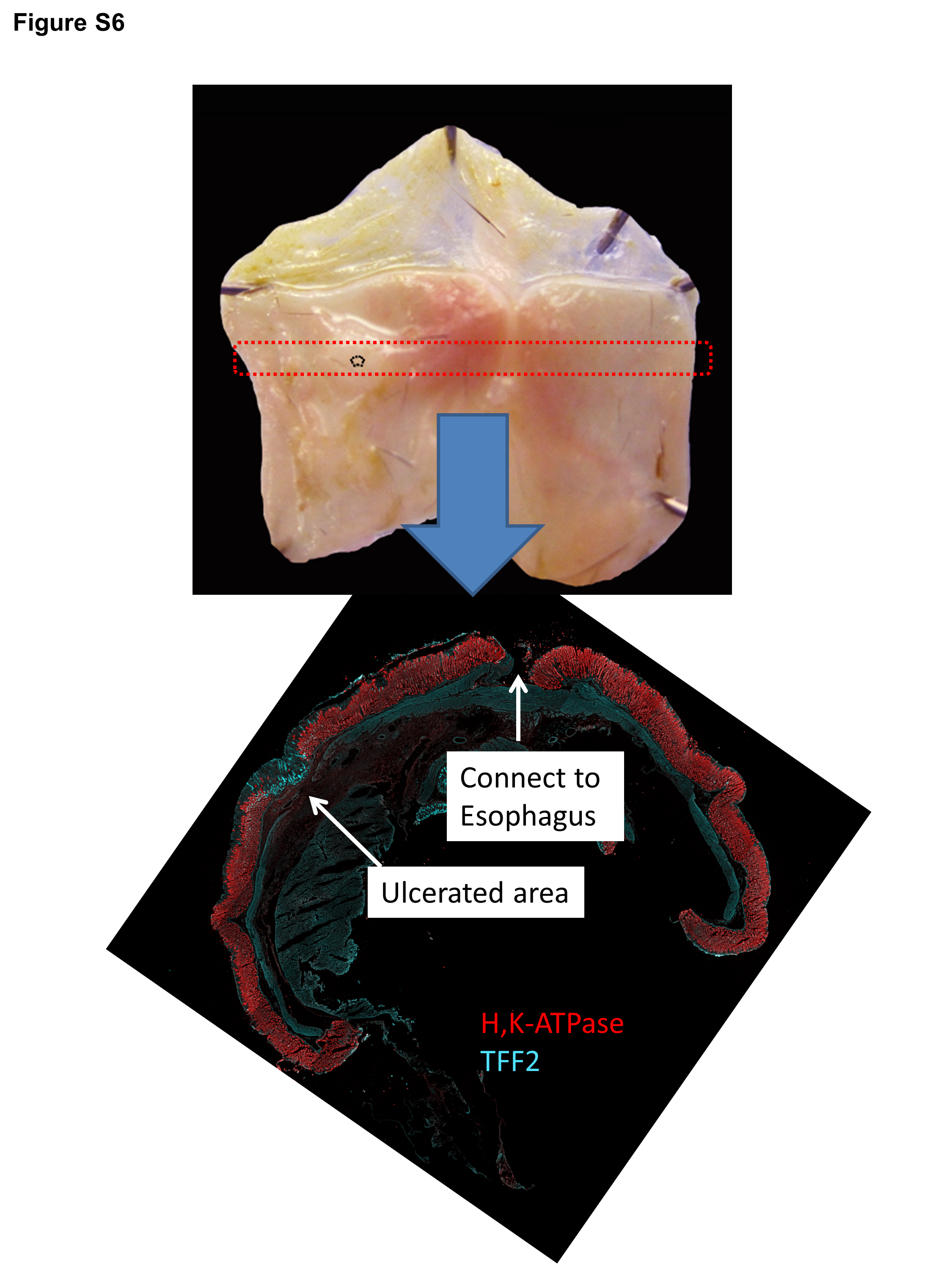

Supplement: Figure S6 — Specific localization of parietal and mucous cell re-distribution caused by ulceration. Gastric ulcer was induced by application of acetic acid as described in Methods, with tissue evaluated 9 days after ulceration. Stereoscopic image (top) taken from 9 days control in Figure 1D, and red rectangle indicates region imaged by confocal microscopy in lower image, evaluating TFF2 (blue) and H,K-ATPase (red) immunostaining. (TIF) [file ppat.1004275.s006.tif]

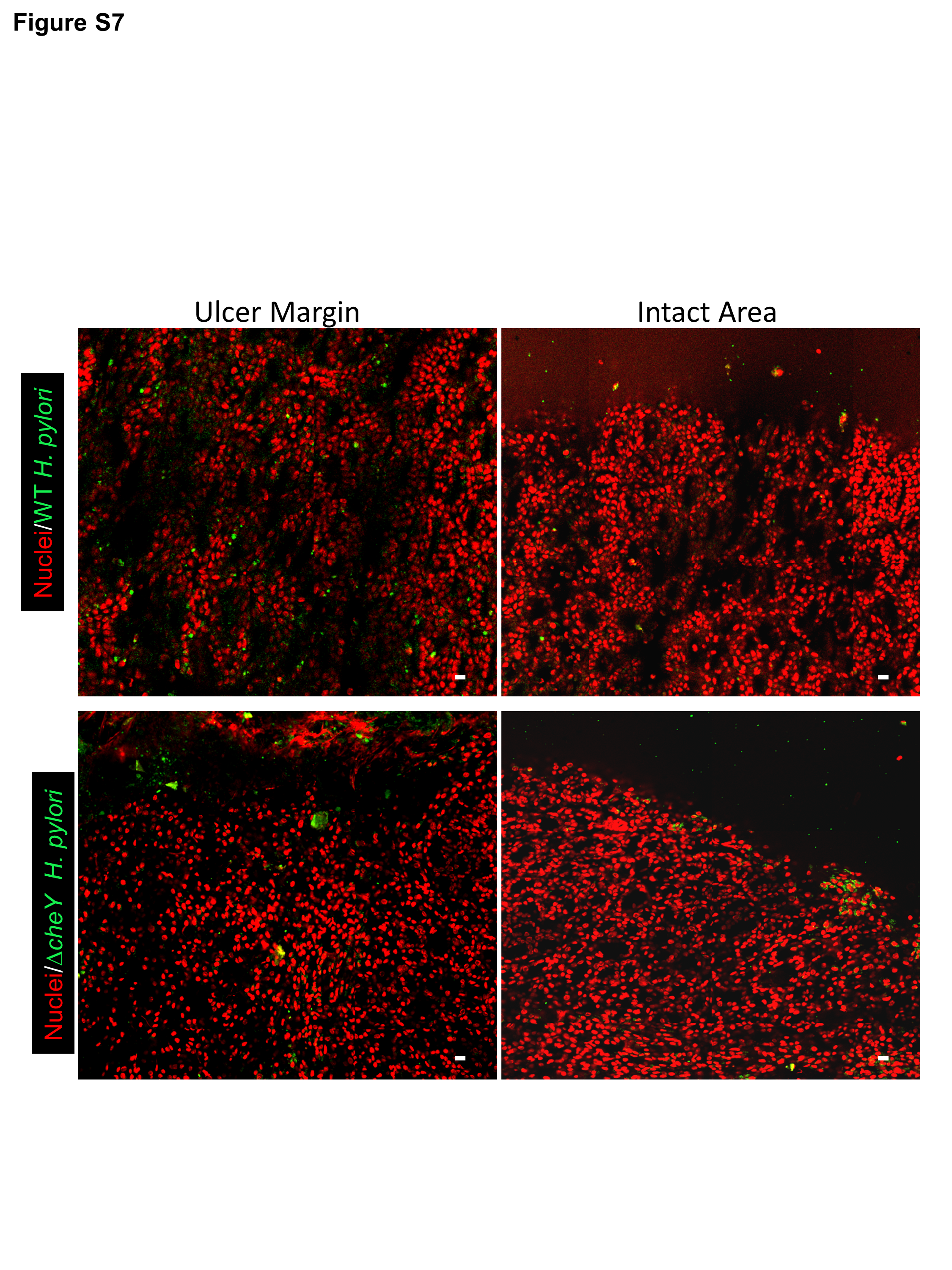

Supplement: Figure S7 — Rapid H. pylori accumulation in the ulcerated area. Gastric ulcer was induced by topical serosal application of acetic acid. Under anesthesia, gastric mucosa was exposed, and placed in the chamber on confocal microscope as described in the Methods. Hoechst 33342 (red) was given intravenously at 5 mg/kg before imaging. Fluorescent labeled wild-type or ΔcheY H. pylori (green) were applied to the gastric mucosa. Confocal microscopy images were taken in the ulcer margin or the non-ulcerated (intact) gastric tissue within 1 hr of applying bacteria. Bar = 10 µm. (TIF) [file ppat.1004275.s007.tif]

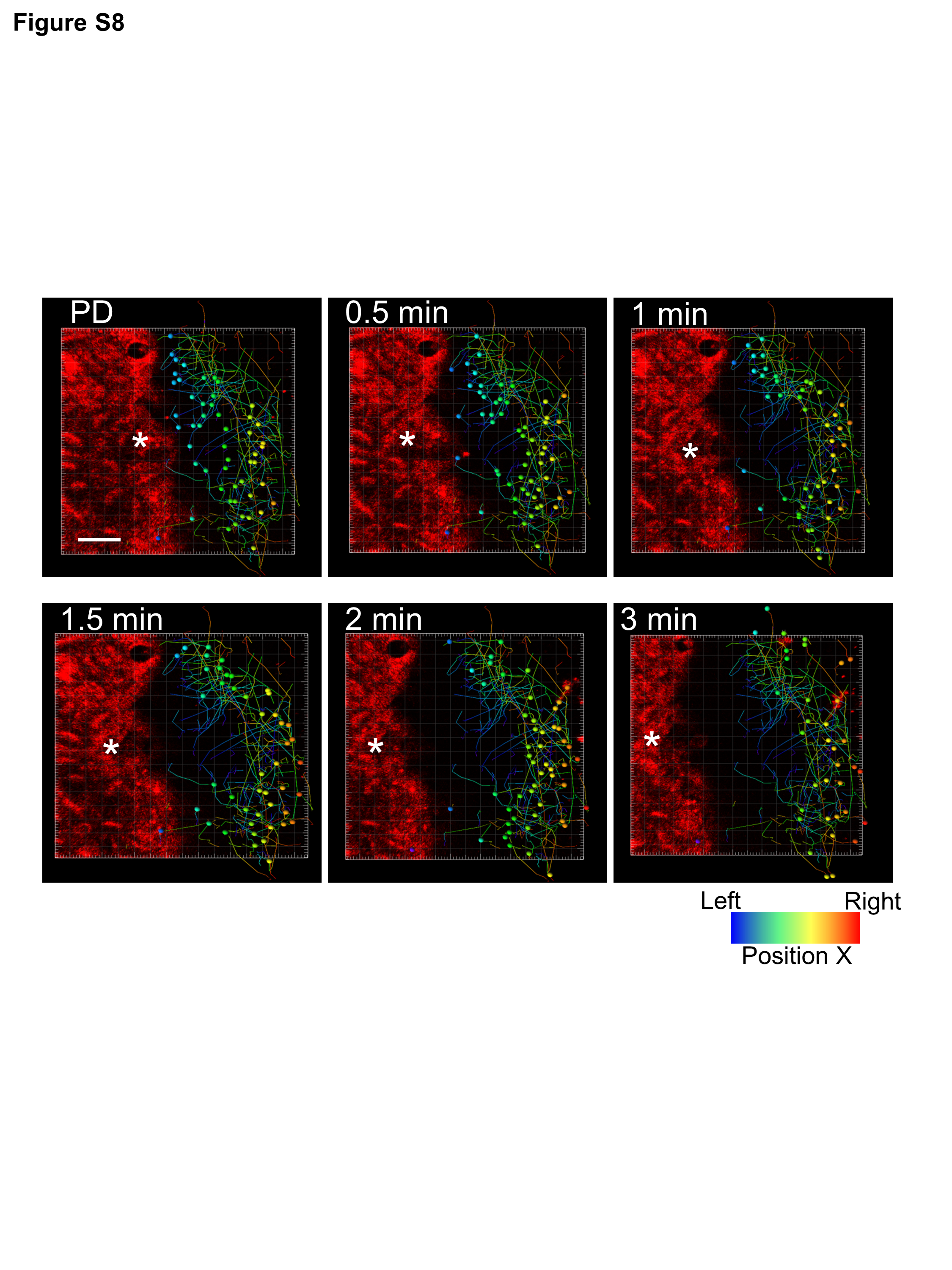

Supplement: Figure S8 — Fluorescent bead movement in the gastric lumen after epithelial damage. 105 fluorescent beads were added to gastric luminal fluid. Two-photon damage was imposed on a microscopic region of the exposed gastric mucosa as described in Methods. Confocal images of time course after photodamage (PD) imposed at time zero. Gastric tissue confocal reflectance (red), bead fluorescence (green). Asterisk indicates site of PD, Image analysis by Imaris 7.6 software (Bitplane) of bead motion over time. Each line tracks an individual bead, pseudo-colored to show progression of distance from tissue over 3 min. Asterisk indicates tissue site of PD. Each line tracks an individual bead, pseudo-colored to show X position on the image window. Bar = 50 µm. (TIF) [file ppat.1004275.s008.tif]

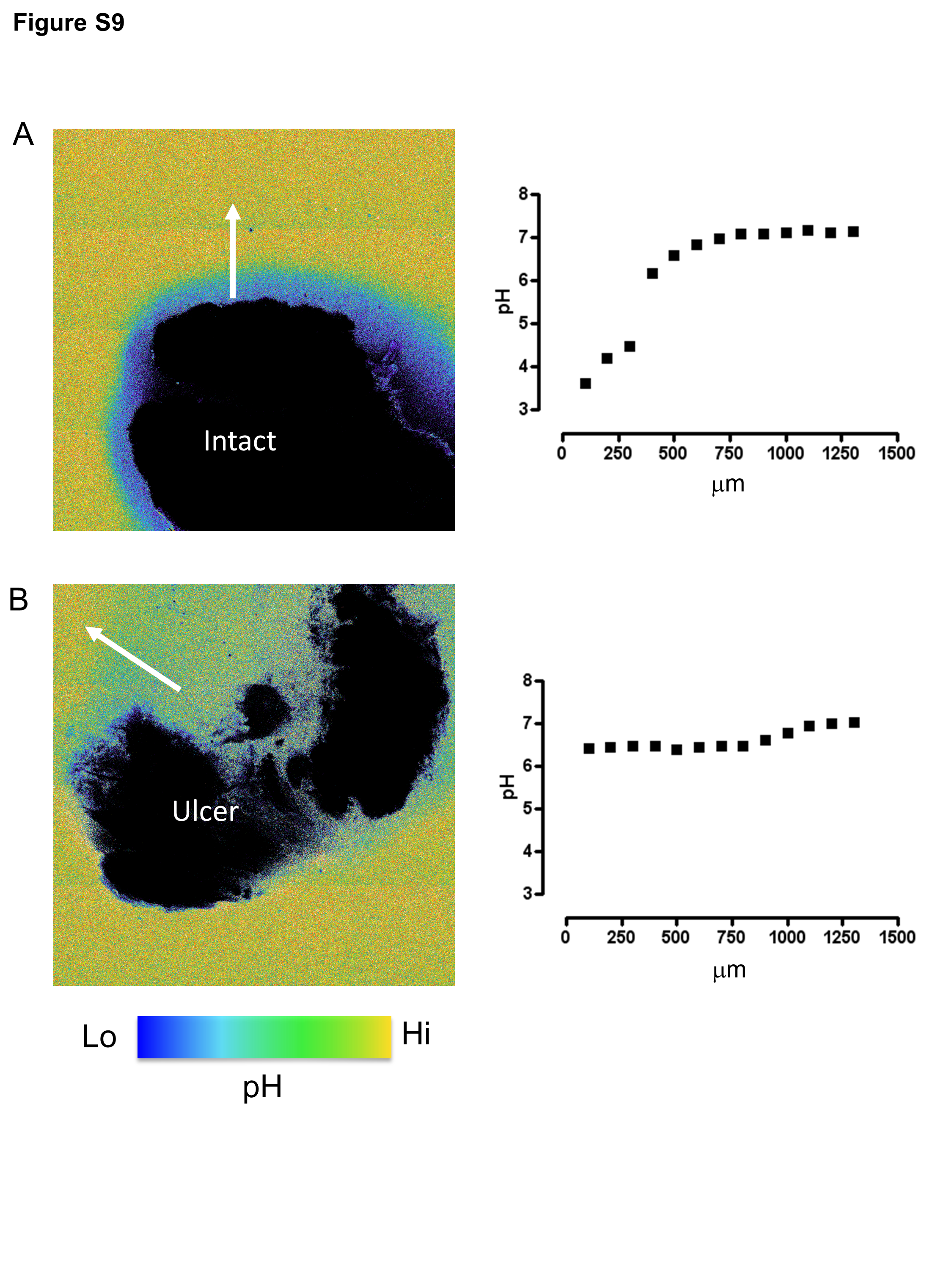

Supplement: Figure S9 — Luminal pH change adjacent to the ulcerated stomach. Gastric ulcer was induced by acetic acid as described in Methods. The mouse was anesthetized and gastric surface surgically exposed for confocal imaging 3 days after ulceration. Gastric lumen was perfused with pH 7 saline containing 20 µM BCECF. BCECF was alternately excited by 488 nm and 458 nm, with 500–550 nm emission. 488 nm/458 nm ratio was fitted to calibration curve to convert to extracellular pH value as described previously [42]. Representative images/analysis shows gastric luminal pH as a function of distance from tissue (position zero) comparing (A) non-ulcerated stomach (intact) area, or (B) ulcerated area. (TIF) [file ppat.1004275.s009.tif]
